# Global, regional, and national burden of intracerebral hemorrhage in adolescents and young adults and its predictions: a systematic analysis of the Global Burden of Disease Study 2021

**DOI:** 10.3389/fneur.2025.1554952

**Published:** 2025-09-08

**Authors:** Guangzhi Hao, Yuwei Han, Yong Liang, Bingying Zhang, Yushu Dong, Guobiao Liang

**Affiliations:** Department of Neurosurgery, General Hospital of Northern Theater Command, Shenyang, Liaoning, China

**Keywords:** intracerebral hemorrhage, Global Burden of Disease, predictions, sociodemographic index, adolescents

## Abstract

**Objective:**

This study analyzes the global, regional, and national burden of intracerebral hemorrhage (ICH) in adolescents and young adults using data from the Global Burden of Disease Study 2021. ICH in younger populations impacts cognitive and physical health, hindering educational and professional development. This research provides insights into ICH trends, burden distribution, and future projections to support targeted public health strategies.

**Methods:**

We extracted age-standardized incidence, mortality, and disability-adjusted life years (DALYs) data for ICH from 1990 to 2021, stratified by age, sex, and Socio-demographic Index (SDI). Estimates were generated using the DisMod-MR 2.1 Bayesian meta-regression framework. Temporal trends were analyzed, and decomposition analysis was performed to quantify the contributions of population growth, aging, and epidemiological changes to the evolving ICH burden. Frontier analysis was used to evaluate the performance of countries relative to their SDI levels. Forecasts of ICH burden through 2044 were produced using the Nordpred age-period-cohort model, with internal validation and sensitivity analyses conducted to assess model robustness.

**Results:**

From 1990 to 2021, global age-standardized ICH incidence, DALYs, and mortality rates declined, though absolute cases, deaths, and DALYs rose in low- and middle-SDI regions. High-SDI areas showed the most substantial burden reductions, while Oceania and Sub-Saharan Africa exhibited higher rates due to limited healthcare resources. Projections suggest further declines in age-standardized DALYs and mortality, though incidence may rise by 2044.

**Conclusion:**

Despite declining age-standardized rates, absolute ICH burdens continue to grow in low-SDI regions, underscoring the need for tailored public health policies and resource allocation to reduce ICH disparities in young populations, especially in underserved regions. Equitable healthcare resources and targeted interventions are essential for reducing global ICH disparities and improving outcomes.

## 1 Introduction

Intracerebral hemorrhage (ICH) is a serious neurological event characterized by bleeding within brain tissue resulting from the rupture of blood vessels (1, 2). ICH is broadly divided into spontaneous and traumatic types, with each category involving complex pathological processes that significantly challenge both diagnosis and treatment (3). Clinically, ICH presents through symptoms such as sudden headaches, altered consciousness, nausea, and hemiplegia, all of which can severely compromise cognitive and physical functions, dramatically reducing quality of life (4). Moreover, ICH carries a substantial risk of disability and mortality, often leaving survivors with long-term impairments that impact not only personal health but also family and community structures (5).

In adolescents and young adults, the consequences of ICH are particularly concerning due to its occurrence at a life stage crucial for educational, personal, and professional development. While advancements in medical imaging and surgical techniques have contributed to improved outcomes in high-income regions, disparities in access to care and early intervention remain prevalent in many low- and middle-income countries, where ICH often leads to severe, unaddressed disabilities (6). Given the impact of ICH on younger populations, a global perspective on its burden through the use of epidemiological and burden of disease studies is essential. Such analyses allow us to identify regional disparities, observe trends over time, and project future impacts, guiding the development of targeted public health policies, preventive strategies, and resources that can mitigate the growing global burden of ICH in adolescents and young adults.

## 2 Methods

### 2.1 Data sources

This investigation utilized data derived from the Global Burden of Disease Study 2021 (GBD 2021), with a targeted focus on ICH among adolescents and young adults (ages 15–39) across a range of countries and regions. The data included comprehensive information on the incidence, disability-adjusted life years (DALYs), and mortality rates associated with ICH, which were further categorized by age, sex, and geographic region. The primary data source, the Global Health Data Exchange (available at https://vizhub.healthdata.org/gbd-results/), enabled access to these extensive global health metrics. Additionally, to explore the association between ICH burden and socioeconomic conditions, we employed the Socio-demographic Index (SDI)-a multidimensional metric combining data on per capita income, mean educational attainment, and fertility rate. This index facilitates an analysis of how variations in socioeconomic development correlate with the global burden of ICH.

### 2.2 Estimation framework

The GBD framework provides standardized estimates of ICH incidence, mortality, and DALYs per 100,000 individuals, which allows for broad comparisons. DALYs, as a comprehensive indicator of disease burden, combine years of life lost (YLLs) due to premature mortality and years lived with disability (YLDs) caused by non-fatal outcomes of ICH. To ensure consistency and comparability across diverse data sources and regions, the GBD study employed DisMod-MR 2.1, a Bayesian meta-regression tool specifically designed to estimate non-fatal health outcomes. DisMod-MR 2.1 integrates heterogeneous data from vital registration, hospital discharge records, and population-based surveys, while adjusting for biases, underreporting, and data sparsity. It applies prior distributions, smoothing across age, time, and location, and propagates uncertainty through Monte Carlo simulation to produce 95% uncertainty intervals for all estimates. To enable age-comparable assessments, all incidence, mortality, and DALY rates were age-standardized using the World Health Organization's standard population, which allows the disentangling of true epidemiologic trends from those driven by demographic structure. Future projections (2022–2044) of the ICH burden were generated using the Nordpred model, an age-period-cohort (APC) forecasting method widely used in global health research. The model was applied using 5-year age groups and periods. The drift component—reflecting the linear trend across successive periods—was attenuated by 25, 50, and 75% for the first, second, and third forecast intervals, respectively, to avoid over-projection. Model fit was evaluated through residual analysis and deviance statistics. To assess the robustness of projections, we conducted an internal validation procedure: we trained the Nordpred model using data up to 2010 and compared forecasts for 2011–2019 against actual GBD estimates. In addition, sensitivity analyses were performed by varying drift attenuation assumptions (0, 50, and 100%).

### 2.3 Statistical analysis

In line with the Global Burden of Disease Study 2021, we calculated the age-standardized incidence, mortality, and DALY rates for ICH, along with 95% confidence intervals (CIs), using the world standard population as a baseline (7). This standardization ensures the estimates are expressed per 100,000 individuals, facilitating consistent comparisons across different age and demographic groups (8). We conducted comparative analyses by sex, age groups (divided into 5-year intervals: 15–19, 20–24, 25–29, 30–34, and 35–39 years), and across the SDI spectrum (five categories: high, high-middle, middle, low-middle, and low SDI levels). The results were consistently reported per 100,000 population to allow for cross-comparison among different regions, age cohorts, and SDI categories. Furthermore, a decomposition analysis was carried out to discern the impacts of factors such as population growth, demographic aging, and shifts in epidemiological trends on the overall disease burden from 1990 to 2021. Statistical significance was defined as a *P*-value < 0.05. The Nordpred method, widely recognized in epidemiological forecasting, was utilized to project disease burden within SDI categories and across 21 global regions (9). Through age-period-cohort analysis, this method enables robust future trend predictions by systematically incorporating historical data and demographic changes to estimate potential global health scenarios. Statistical analyses were performed using R software (version 4.4.2), with results deemed significant at *P* < 0.05.

## 3 Results

### 3.1 Temporal trend of intracerebral hemorrhage in the adolescents and young adults from 1990 to 2021

Globally, the age-standardized incidence rate, DALYs, and mortality rate decreased from 11.85 (95% CI: 7.96–16.69), 271.00 (246.95–293.66), and 4.30 (3.91–4.66) in 1990 to 8.14 (5.65–11.31), 177.45 (159.88–196.14), and 2.79 (2.51–3.10) in 2021, respectively (Supplementary Table 1). Furthermore, we observed that females had lower age-standardized incidence, DALYs, and mortality rates compared to males, with this difference being particularly notable in the 30–39 age group (Supplementary Table 2).

**Figure 1:**
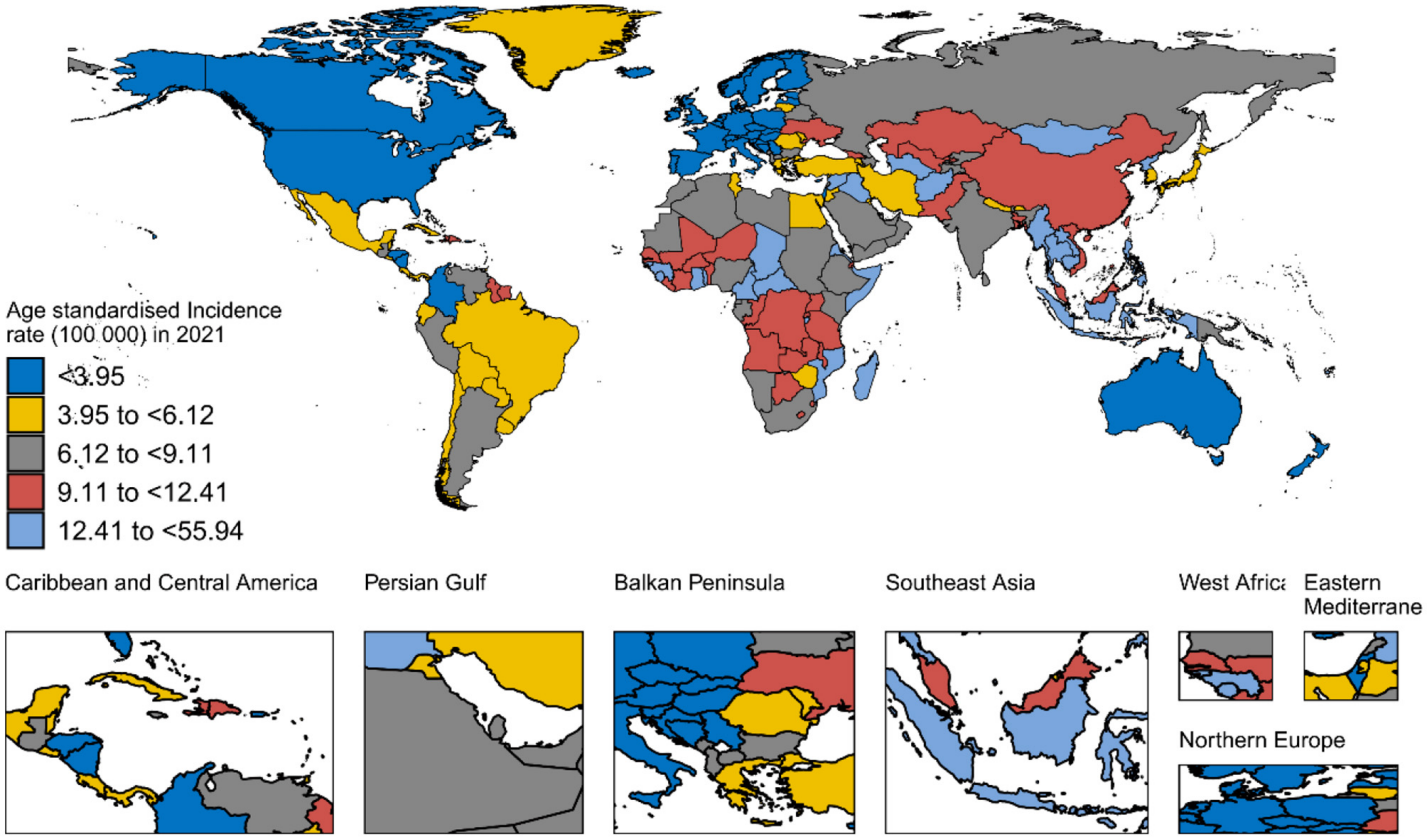
Geographical distribution of age-standardized incidence.

In 2021, countries with the lowest age-standardized incidence, DALYs, and mortality rates included high-SDI regions such as Switzerland, New Zealand, Israel, Australia, and Norway. Conversely, countries with the highest age-standardized incidence rates, DALYs, and mortality rates were predominantly in low-middle and middle-SDI regions, such as Kiribati, Nauru, the Marshall Islands, Vanuatu, and Micronesia (Federated States of; Figure 1, and Supplementary Table 3).

**Figure 2:**
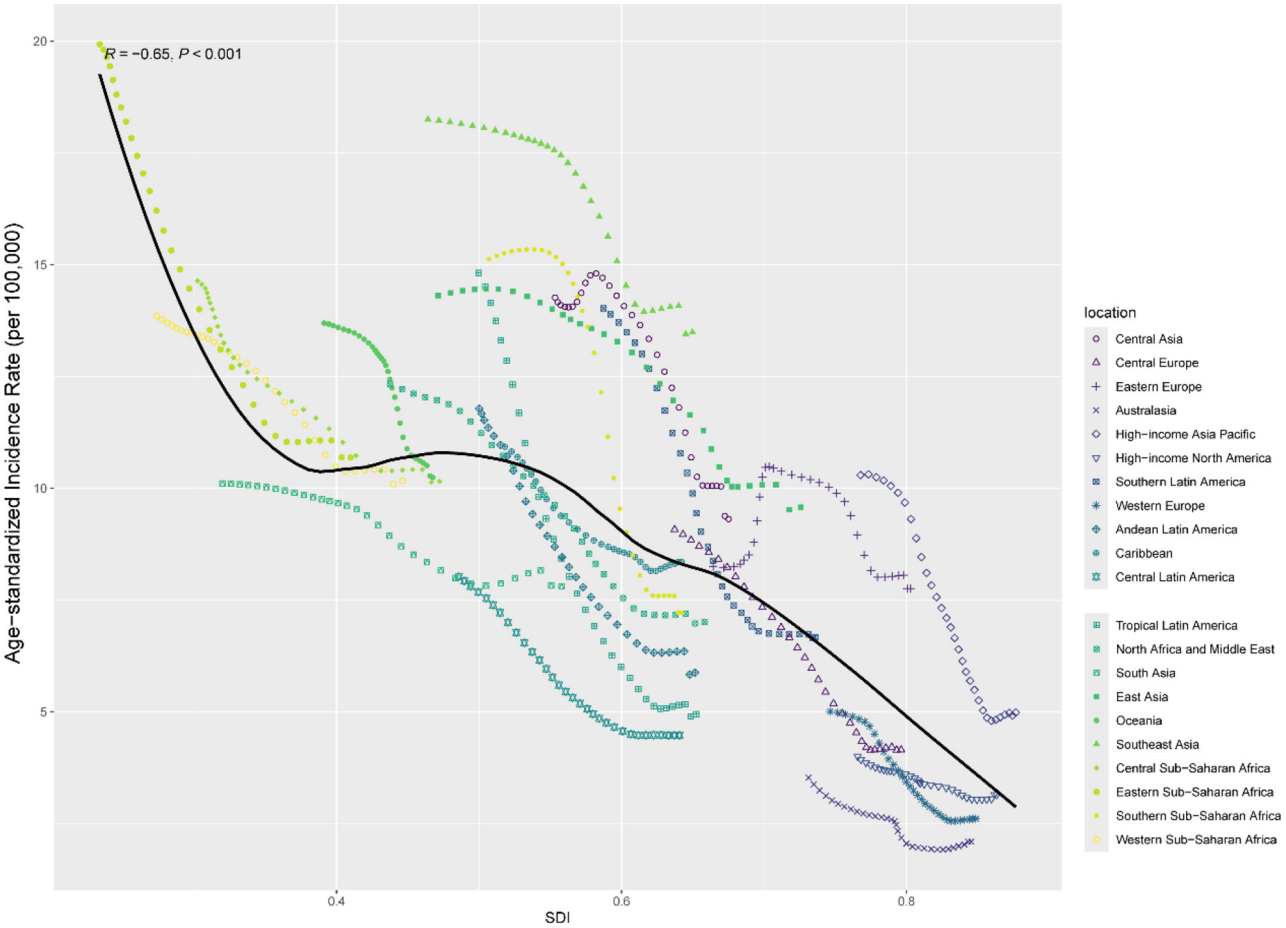
Age-standardized incidence for intracerebral hemorrhage by SDI, 1990–2021, and expected value-based SDI.

Figure 2 and Supplementary Figures 1, 2 illustrate the smoothed curves for age-standardized incidence rates, DALYs, and mortality rates across 21 regions. Using a smoothed spline model (*R* = −0.65, −0.58, −0.59, respectively; all *P* < 0.001), we found a clear decreasing trend in age-standardized incidence, DALYs, and mortality rates with increasing SDI. The highest age-standardized incidence rates were observed in Southeast Asia, Eastern Sub-Saharan Africa, Oceania, and Western Sub-Saharan Africa, while the lowest incidence rates were found in Australasia, Western Europe, High-income North America, and Central Europe.

Supplementary Figures 3–5 display age-standardized incidence, DALYs, and mortality rates for 204 countries. Through a smoothed spline model, we observed a decline in age-standardized incidence, DALYs, and mortality rates as SDI increased (ρ = −0.67, −0.74, −0.75; all *P* < 0.001). The age-standardized incidence, DALYs, and mortality rates in Kiribati, Nauru, the Solomon Islands, the Marshall Islands, Vanuatu, Micronesia (Federated States of), Palau, and Tuvalu were notably higher than the smoothed curve.

### 3.2 Decomposition analysis

**Figure 3:**
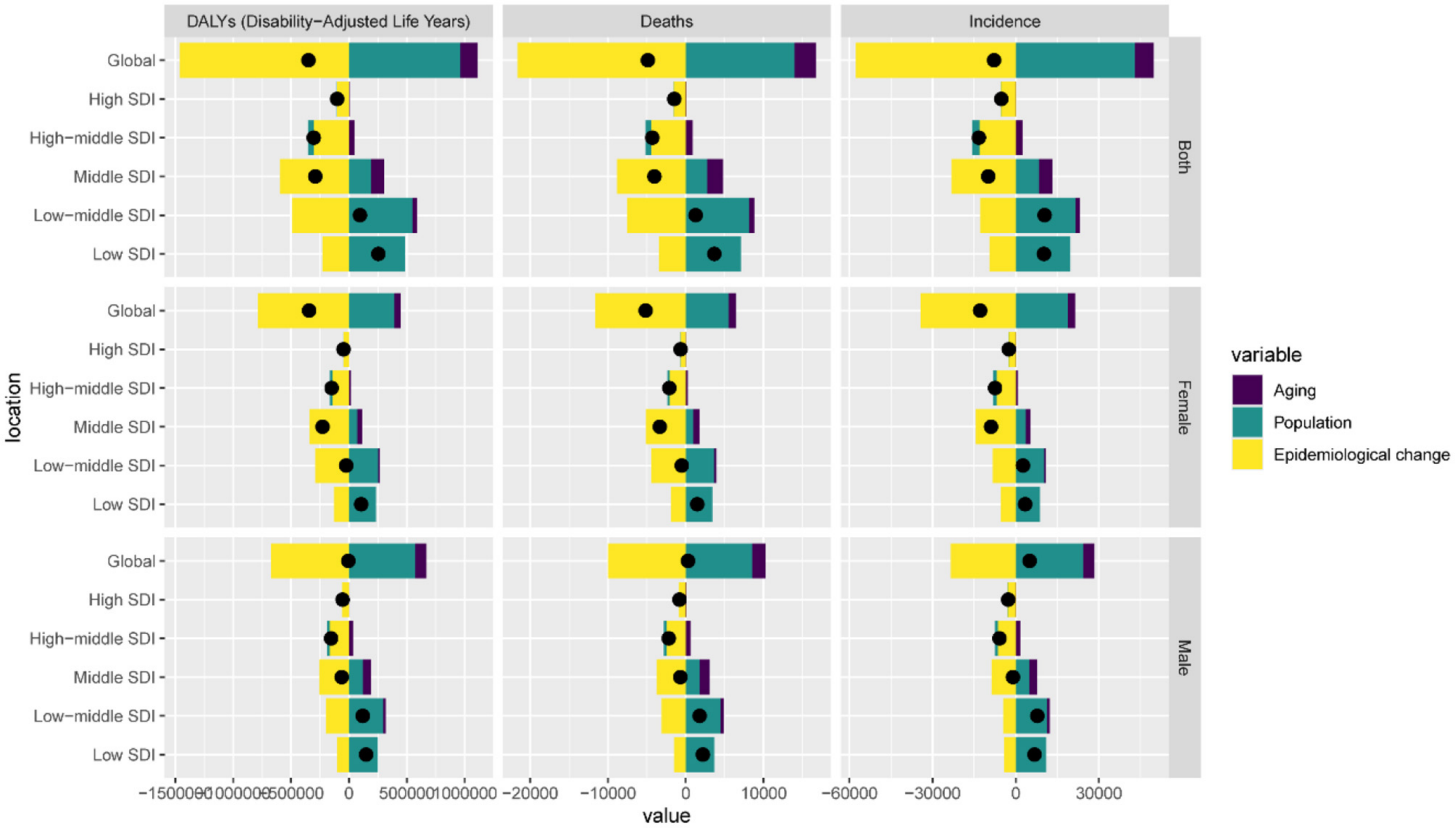
Changes in intracerebral hemorrhage DALYs, deaths and incidence according to population-level determinants of population growth, aging, and epidemiological change from 1990 to 2021 at the global level and by SDI.

Figure 3 presents the decomposition of changes in DALYs, deaths, and incidence of ICH from 1990 to 2021, stratified by SDI region and sex. At the global level, epidemiological changes were the dominant driver of burden increase across all three indicators, accounting for 417.8% of the change in DALYs, 443.1% in deaths, and 738.8% in incidence. In contrast, both population aging and growth contributed negatively to these changes. Notable heterogeneity was observed across SDI regions. In high and high-middle SDI countries, epidemiological changes were the primary drivers of the increased disease burden in both males and females, while the impact of population aging was limited and even offset the increase in burden. In low-SDI regions, the influence of population growth was substantial. However, epidemiological changes showed a slight mitigating effect on the disease burden. In middle-SDI regions, epidemiological changes were the primary drivers of increased ICH burden, while population growth and aging had a mitigating effect. In low-middle-SDI regions, population growth emerged as the dominant factor, however, these increases were partially offset by substantial negative contributions from epidemiological change. For females, the pattern was reversed—epidemiological changes led to sharp increases, while aging and population growth had negative or minor effects.

### 3.3 Frontier analysis

**Figure 4:**
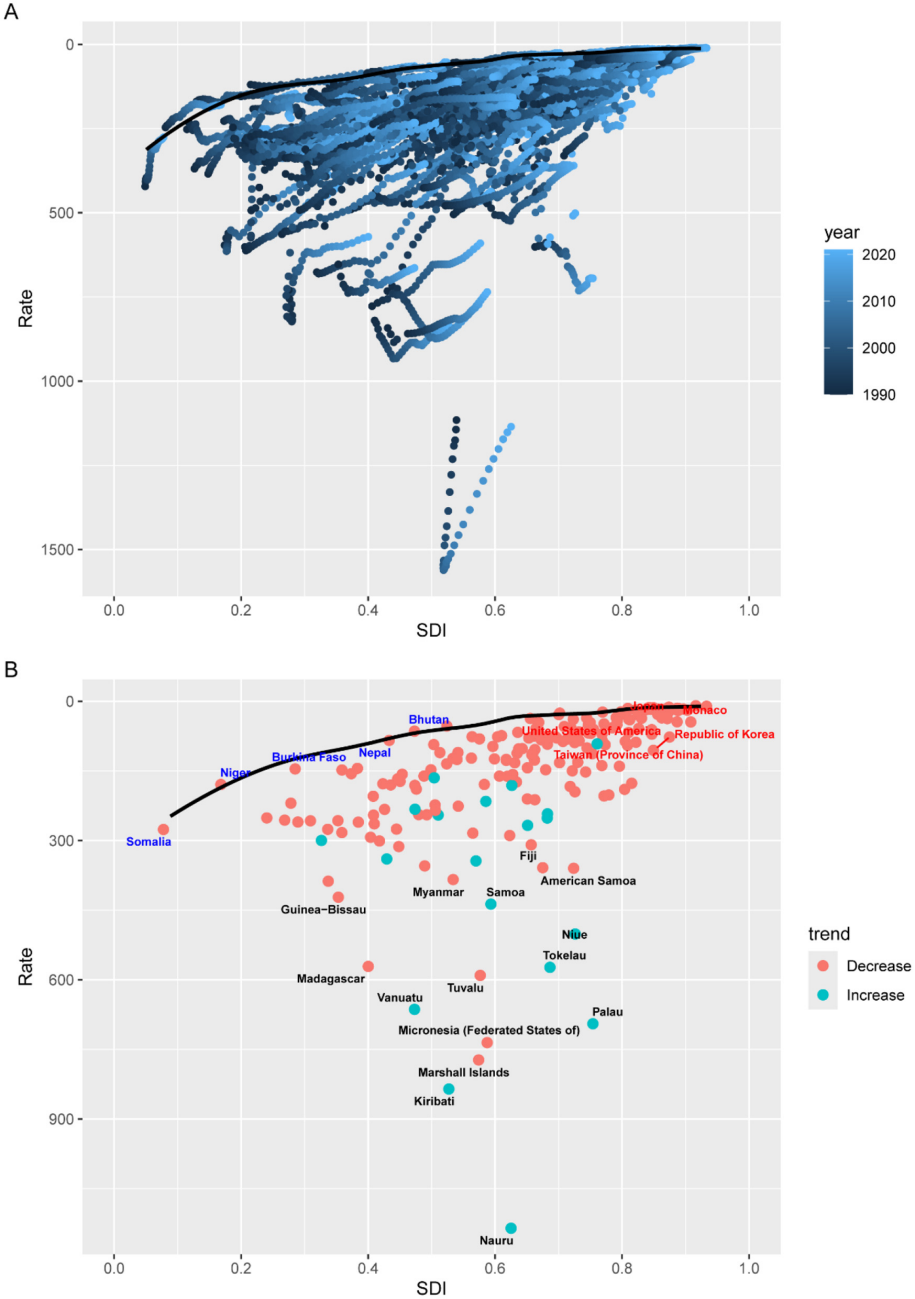
Frontier analysis on the basis of SDI and age-standardized DALYs per 100,000 of intracerebral hemorrhage from 1990 to 2021. **(A)** 1990–2021; **(B)** 2021.

Figure 4 presents the frontier analysis for age-standardized DALY rates of ICH among adolescents and young adults across 204 countries and territories from 1990 to 2021. Figure 4A illustrates the global frontier curve, highlighting how, at each SDI level, certain countries have achieved relatively low ICH burdens. Countries lying closer to the frontier line demonstrate more efficient management or prevention of ICH among youth, while those farther away indicate substantial room for burden reduction. Figure 4B focuses on 2021 and plots all countries according to their distance from the frontier. Red dots indicate countries where age-standardized DALY rates declined between 1990 and 2021, while blue dots represent countries with increasing rates. Countries labeled in red font highlight the five high-SDI countries with the most inefficient performance relative to their development level. These include Japan, Monaco, United States of America, Republic of Korea and Taiwan (Province of China)—all of which maintain advanced healthcare infrastructure yet continue to face high burden in youth ICH, possibly due to behavioral risks, trauma incidence, or delays in early cerebrovascular intervention. Conversely, countries labeled in blue font represent the five low-SDI countries with the smallest gap to the frontier. These include Somalia, Niger, Burkina Faso, Nepal and Bhutan, where DALY rates in young populations closely approached their SDI-based frontier. Despite limited resources, their relative efficiency may reflect successful grassroots healthcare initiatives. Together, these findings underscore profound disparities in burden control efficiency across countries and development levels.

### 3.4 Attributable risk factors for DALYs in ICH among adolescents and young adults

**Figure 5:**
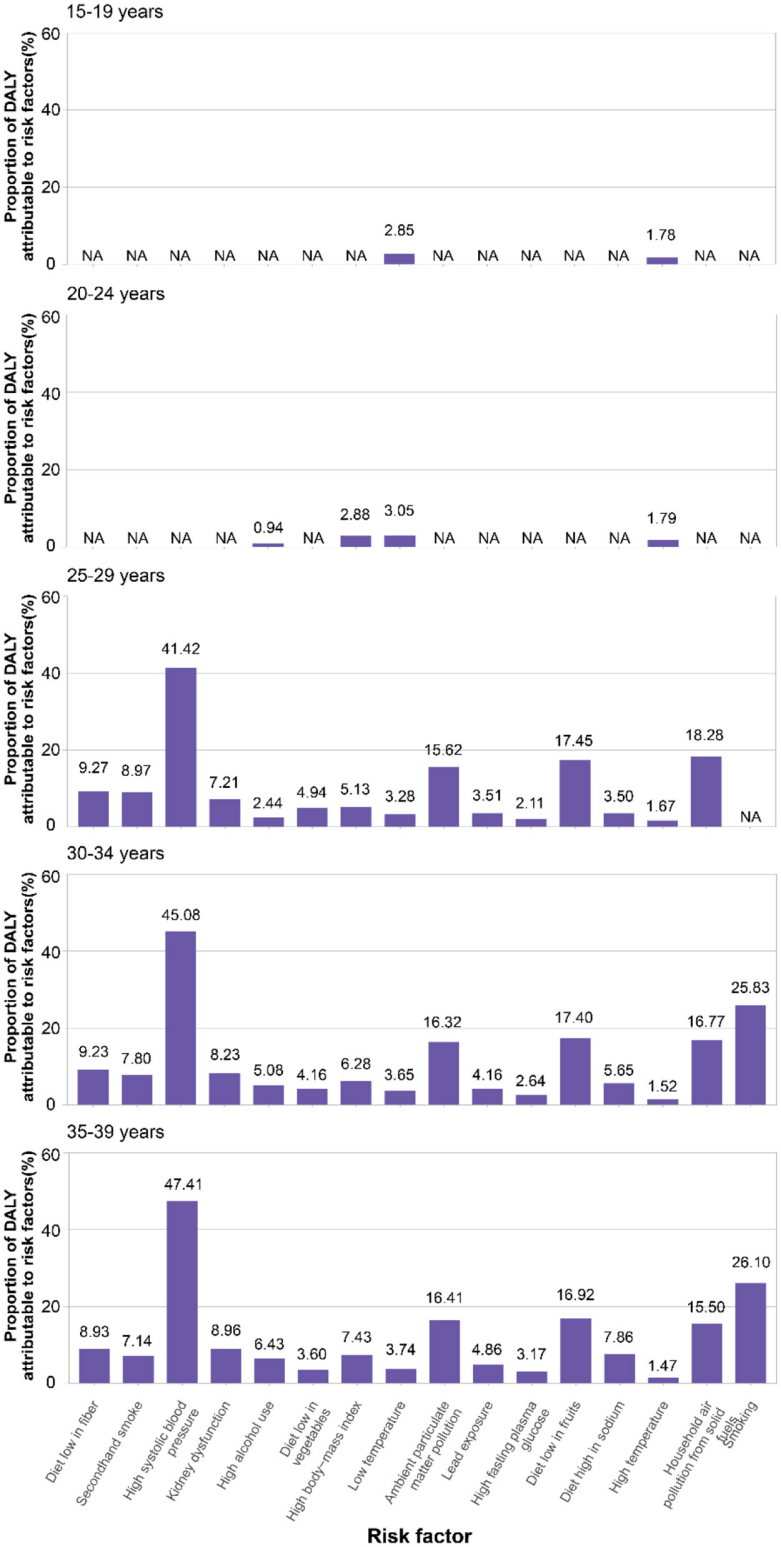
Proportion of intracerebral hemorrhage DALY in adolescents and young adults attributable to 16 risk factors in 2021.

The contribution of specific risk factors to DALYs due to ICH varied markedly across different age groups among adolescents and young adults, reflecting distinct patterns of environmental, behavioral, and metabolic exposures during early life stages (Figure 5). In the 15–19 years age group, climate-related exposures were the sole attributable contributors. Low temperature accounted for 2.85%, and high temperature for 1.78% of ICH-related DALYs, respectively. No behavioral, dietary, or metabolic risk factors were identified in this cohort, suggesting a unique vulnerability of adolescents to extreme ambient environmental conditions. Among individuals aged 20–24 years, the impact of modifiable behavioral and metabolic risk factors began to emerge. Low temperature remained the predominant contributor to ICH-related DALYs (3.05%) in this age group, followed by elevated body mass index (2.88%), high ambient temperature (1.79%), and alcohol use (0.94%). These patterns suggest a transitional phase in early adulthood during which environmental exposures begin to converge with emerging behavioral and metabolic risk factors, reflecting a shift toward modifiable lifestyle determinants of cerebrovascular health. In the 25–29 years age group, the attributable burden of ICH exhibited a notably complex and multifactorial profile. High systolic blood pressure emerged as the leading contributor, accounting for 41.42% of total DALYs, underscoring the early onset of hypertensive disease burden in young adulthood. Beyond metabolic influences, environmental and dietary risk factors played a substantial role. Household air pollution from solid fuels contributed 18.28% of DALYs, followed closely by insufficient fruit intake (17.45%) and ambient particulate matter pollution (15.62%). Other modifiable risks such as low dietary fiber (9.27%), secondhand smoke exposure (8.97%), and kidney dysfunction (7.21%) also demonstrated considerable impacts. Among individuals aged 30–34 years, high systolic blood pressure remained the predominant risk factor and further increased its attributable share to 45.08% of ICH-related DALYs, consolidating its status as the most critical modifiable factor in early middle age. Smoking rose sharply to become the second most impactful risk, contributing 25.83% of DALYs. Dietary insufficiencies and environmental exposures remained significant, with a diet low in fruits accounting for 17.40%, household air pollution for 16.77%, and ambient particulate matter pollution for 16.32%. In the 35–39 years, the burden of ICH attributable to high systolic blood pressure continued to rise, reaching 47.41% of total DALYs, reflecting the cumulative effect of prolonged hypertensive exposure. Smoking maintained its position as the second leading risk factor with an attributable fraction of 26.10%, further emphasizing the need for intensified tobacco control strategies. Diet-related risks remained substantial, with low fruit intake (16.92%) and low fiber intake (8.93%) demonstrating continued relevance. Concurrently, ambient particulate matter pollution (16.41%) and household air pollution from solid fuels (15.50%) persisted as major environmental hazards. Metabolic dysfunctions such as kidney impairment (8.96%) and high BMI (7.43%), along with high sodium intake (7.68%) and alcohol use (6.43%), also contributed meaningfully to disease burden. Compared with younger cohorts, the 35–39 years group exhibited an amplified accumulation of multiple interacting risk factors, suggesting that early middle age is a critical window for multifaceted intervention targeting cardiovascular health, environmental exposure, and modifiable behaviors.

### 3.5 Burden of disease projections

**Figure 6:**
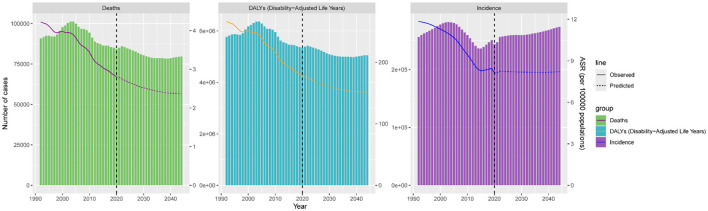
Age-standardized deaths, DALYs, and incidence for global intracerebral hemorrhage for the observational period (1990–2021) and the projection period (2022–2044).

Using the Nordpred model, we forecasted the global burden of ICH among adolescents and young adults from 2022 to 2044 (Figure 6, Supplementary Tables 4–6). The global ASMR for ICH is projected to decrease steadily from 2.75 per 100,000 in 2022 to 2.37 per 100,000 in 2044. Despite this overall decline, the sex disparity is expected to remain stable throughout the projection period. In 2022, the ASMR among males is estimated at 3.62 per 100,000, considerably higher than the 1.94 per 100,000 observed in females. By 2044, these rates are projected to decline to 3.05 and 1.62 per 100,000, respectively. The ASDR is anticipated to show a modest downward trend, decreasing from 174.63 per 100,000 in 2022 to 151.51 per 100,000 in 2044. The ASDR for males is projected to decline from 219.24 per 100,000 in 2022 to 191.66 per 100,000 in 2044, while for females, it is expected to fall from 129.18 to 110.02 per 100,000. The ASIR is projected to remain relatively stable, with a slight decrease from 8.25 per 100,000 in 2022 to 8.21 per 100,000 in 2044. Sex-specific trends show divergent patterns: among males, the ASIR is expected to decrease from 9.92 to 9.72 per 100,000, whereas among females, it is projected to rise slightly from 6.54 to 6.69 per 100,000 over the same period.

## 4 Discussion

This study provides a comprehensive analysis of global and regional trends in the burden of ICH among adolescents and young adults aged 15–39 years from 1990 to 2021, with projections to 2044. Our findings reveal a significant and persistent global burden in this population, with notable heterogeneity across regions, age groups, and SDI levels. Importantly, this study highlights the unique vulnerability of the adolescents and young adults, whose ICH etiology and risk profiles differ substantially from older adults. Traumatic brain injury—stemming from motor vehicle accidents, sports, or interpersonal violence—remains a leading contributor to ICH among younger age groups (10, 11). In addition, the prevalence of substance abuse (including cocaine and amphetamines) and heavy alcohol consumption, both of which are independently associated with increased ICH risk, is disproportionately higher in this demographic (12, 13). Moreover, congenital and genetic factors—such as arteriovenous malformations, aneurysms, and cavernous malformations—are more prevalent causes of ICH in adolescents and young adults compared to older populations (14–16). Unlike hypertensive hemorrhages predominant in the elderly, vascular anomalies and coagulopathies represent key etiological distinctions in younger individuals. These conditions often present with catastrophic hemorrhages, underscoring the need for early screening and tailored preventive strategies in high-risk populations.

From a regional perspective, Sub-Saharan Africa and Oceania exhibited the highest age-standardized mortality and DALY rates attributable to non-communicable neurological disorders among adolescents and young adults. In addition to limited healthcare infrastructure, these regions face a disproportionate burden due to a high prevalence of untreated hypertension, endemic infectious diseases predisposing to vascular injury, and suboptimal trauma care systems (17). For instance, in resource-limited settings, delayed access to imaging, lack of neurosurgical services, and inadequate rehabilitation exacerbates poor neurological outcomes. Notably, household air pollution from solid fuels remains a major environmental risk factor in these regions, with Sub-Saharan Africa having the highest proportion globally, followed by Oceania (18).

Decomposition analysis further revealed that population growth and epidemiologic changes, rather than aging, were the main drivers of increased ICH burden in AYAs. This contrasts with older age groups where demographic aging is dominant, again highlighting the need for age-specific health policies. Our findings from frontier analysis indicate that many low- and middle-SDI regions continue to underperform in reducing DALY rates relative to their developmental potential, emphasizing inefficiencies in health system resource allocation (19). The SDI, a composite measure reflecting the level of development of a country or region, encompasses key dimensions including per capita income, years of education, and total fertility rate. A higher SDI is typically associated with better healthcare resources, higher health awareness, and more comprehensive disease prevention strategies. With increasing SDI, the mortality and DALY rates in most of the 21 regions have shown a downward trend, with particularly pronounced declines observed in Southern Latin America, Tropical Latin America, and Central Europe. This trend may be attributed to ongoing socioeconomic development in these regions, where improvements in treatment technology and disease awareness have enhanced outcomes for ICH patients. According to Xu et al. (19), regions with higher SDI levels exhibit lower ICH DALY and mortality rates; in fact, age-standardized DALY rates in high-SDI regions decreased by as much as 67%, while in low-SDI regions the reduction was relatively modest, at only around 20%−30%. However, countries and regions such as Nauru, Kiribati, the Marshall Islands, and Micronesia (Federated States of) continue to report high mortality and DALY rates, indicating gaps in ICH prevention and treatment efforts that warrant urgent attention.

Age stratification revealed meaningful distinctions in ICH burden and risk exposure patterns among adolescents and young adults. Adolescents aged 15–19 years exhibited the lowest age-standardized DALY rates. In adolescents aged 15–19 years, exposure to high temperature or low temperature emerged as notable contributors to ICH-related DALYs. This pattern highlights the heightened vulnerability of adolescents and young adults to environmental stressors, such as extreme temperatures. These findings are particularly relevant in the context of increasing global climate variability. The observed association underscores the importance of targeted public health strategies, including early warning systems for heat and cold waves and infrastructure improvements to ensure thermal safety in vulnerable populations. In contrast, individuals aged 25–39 years demonstrated increasing susceptibility to modifiable lifestyle-related risk factors, such as high systolic blood pressure, smoking, and diets low in fruit. Notably, our risk factor analysis identified high systolic blood pressure as the leading contributor to ICH across the older segments of the adolescents and young adults, with a rising prevalence of smoking and inadequate fruit intake among both males and females aged 30–39 years.

Future projections indicate that, despite anticipated declines in the global age-standardized DALY and mortality rates for ICH, the incidence rate is expected to increase by 2044. In conclusion, this study demonstrates that, although age-standardized rates of the global ICH burden have decreased, absolute numbers of cases and deaths continue to rise in low-SDI regions. Thus, public health policies and resource allocation should focus more on adolescents and young adults in low-SDI regions. Through global and regional collaboration, prevention efforts and early interventions can reduce the ICH burden in this population, ultimately diminishing disparities and inequities in the global disease burden (20, 21).

## Data Availability

The original contributions presented in the study are included in the article/Supplementary material, further inquiries can be directed to the corresponding authors.
