# Supplementary material for: Global, regional, and national burden of intracerebral hemorrhage in adolescents and young adults and its predictions: a systematic analysis of the Global Burden of Disease Study 2021
Source: Front Neurol. 2025 Sep 8;16:1554952. doi: 10.3389/fneur.2025.1554952 (PMC12450654; doi:10.3389/fneur.2025.1554952)
Supplement: Supplementary file 1 [file Data_Sheet_1.docx]

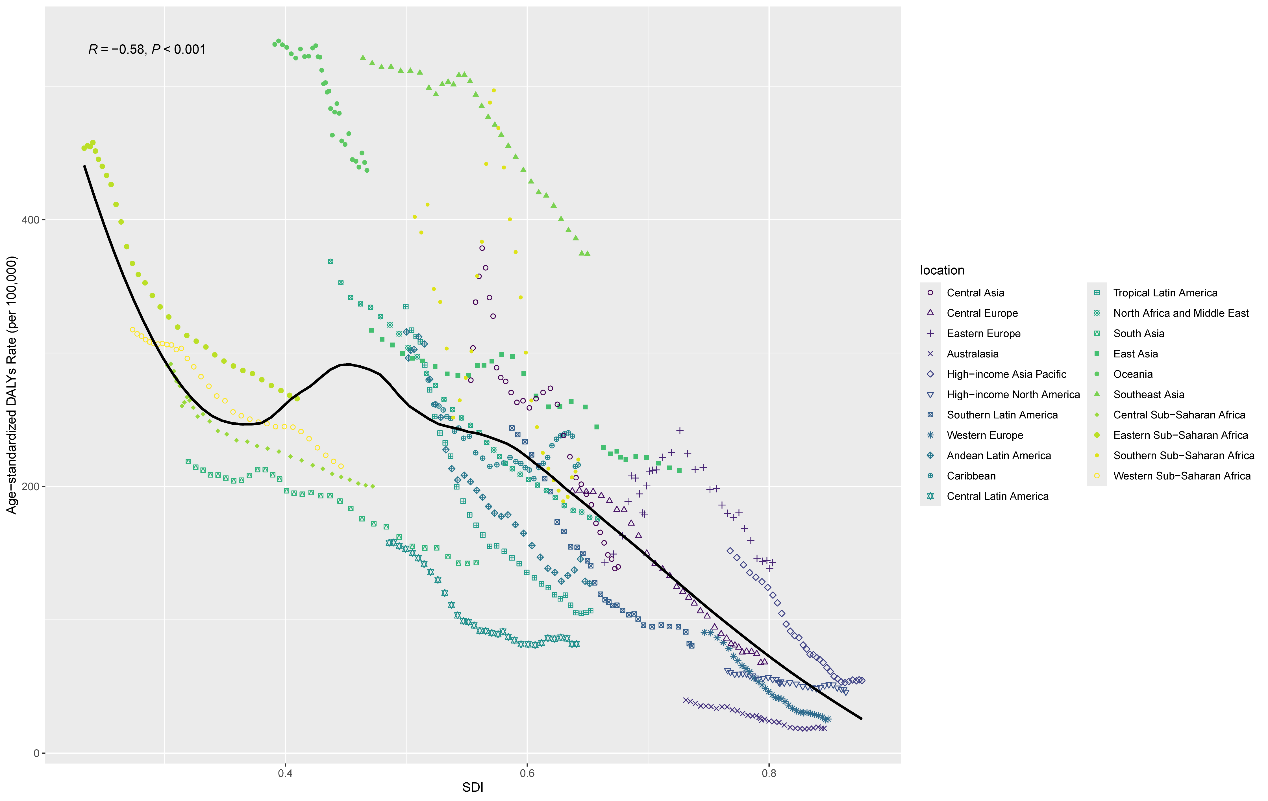


Supplementary Figure 1. Age-standardized disability-adjusted life years (DALYs) for intracerebral hemorrhage by Socio-demographic Index (SDI), 1990-2021, and expected value-based SDI.


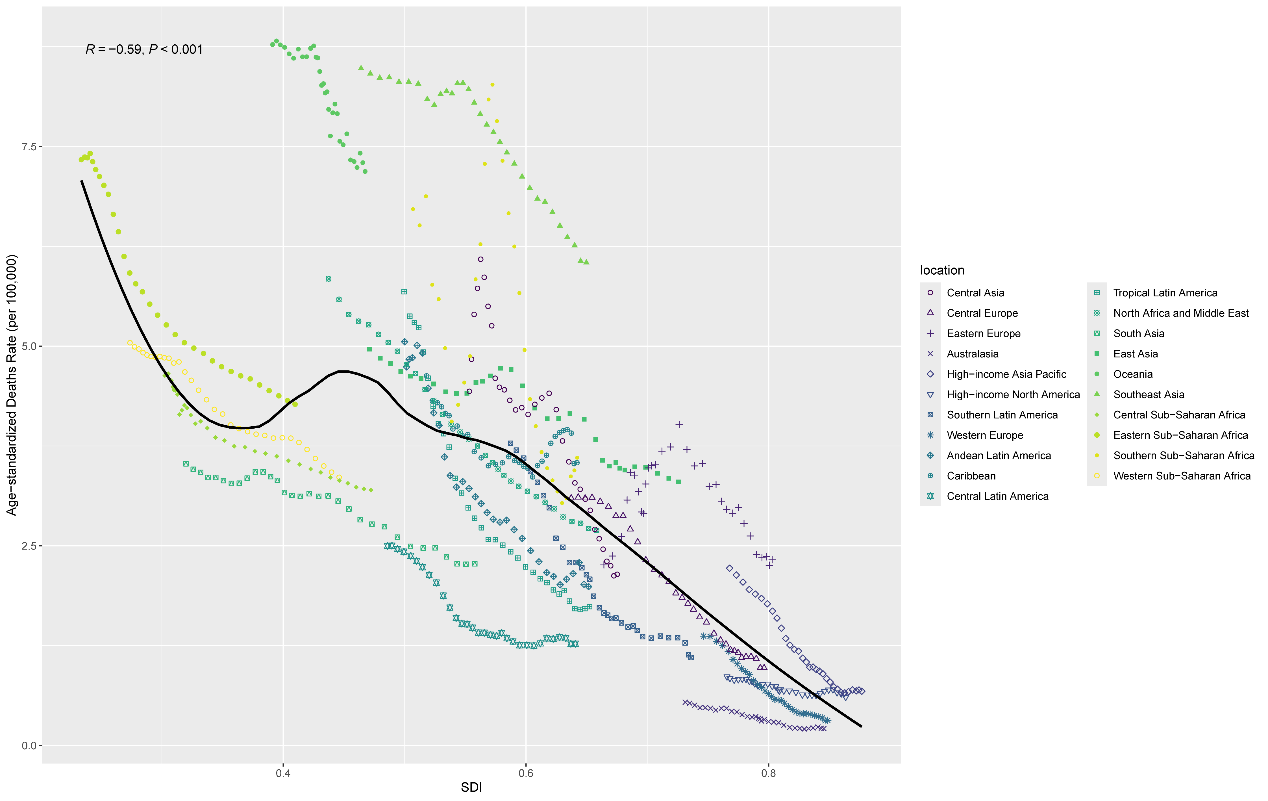


Supplementary Figure 2. Age-standardized mortality for intracerebral hemorrhage by Socio-demographic Index (SDI), 1990-2021, and expected value-based SDI.


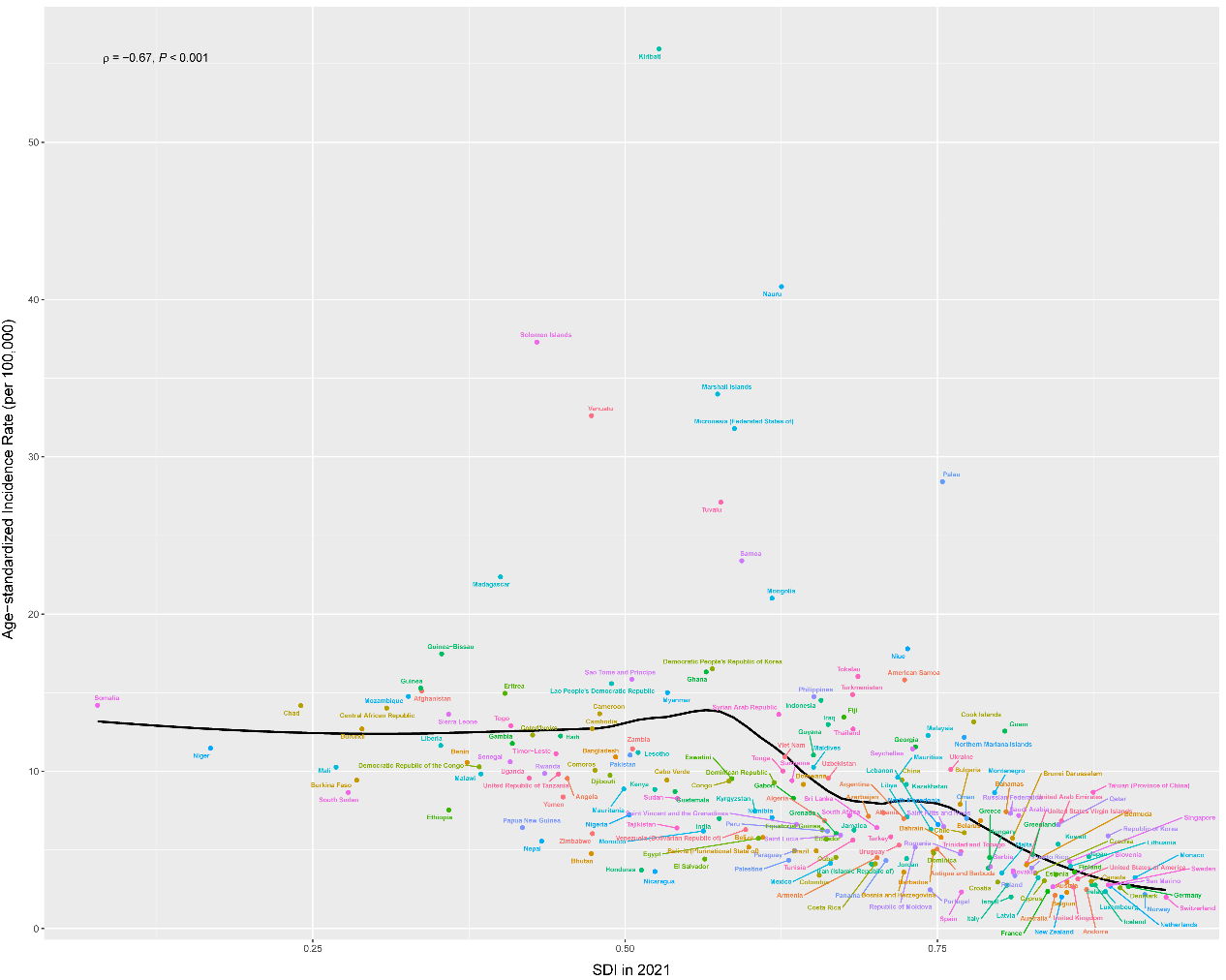


Supplementary Figure 3. Incidence rate of IHC by 204 countries and territories and SDI in 2021.


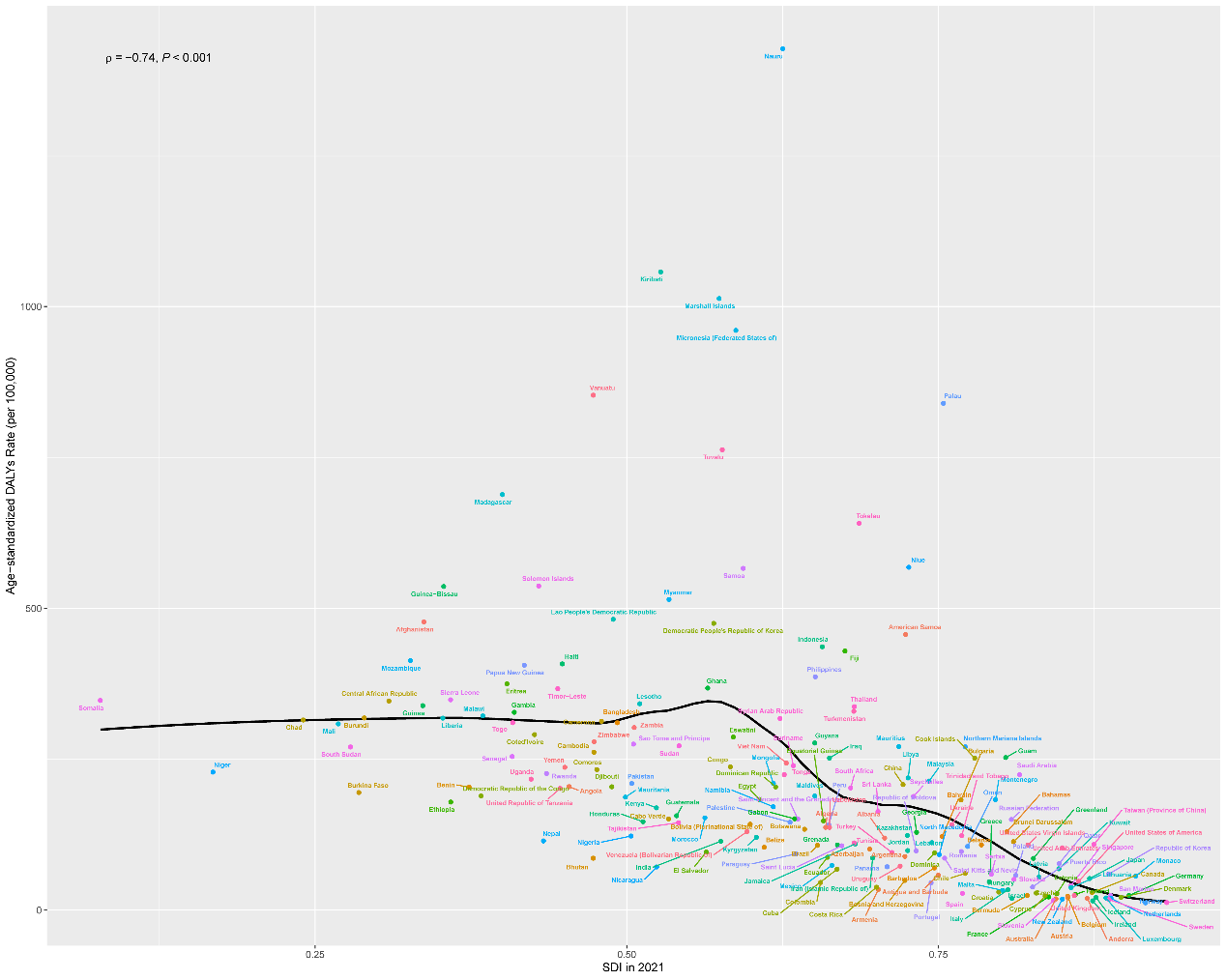


Supplementary Figure 4. Disability-adjusted life years (DALYs) rate of intracerebral hemorrhage by 204 countries and territories and Socio-demographic Index in 2021.


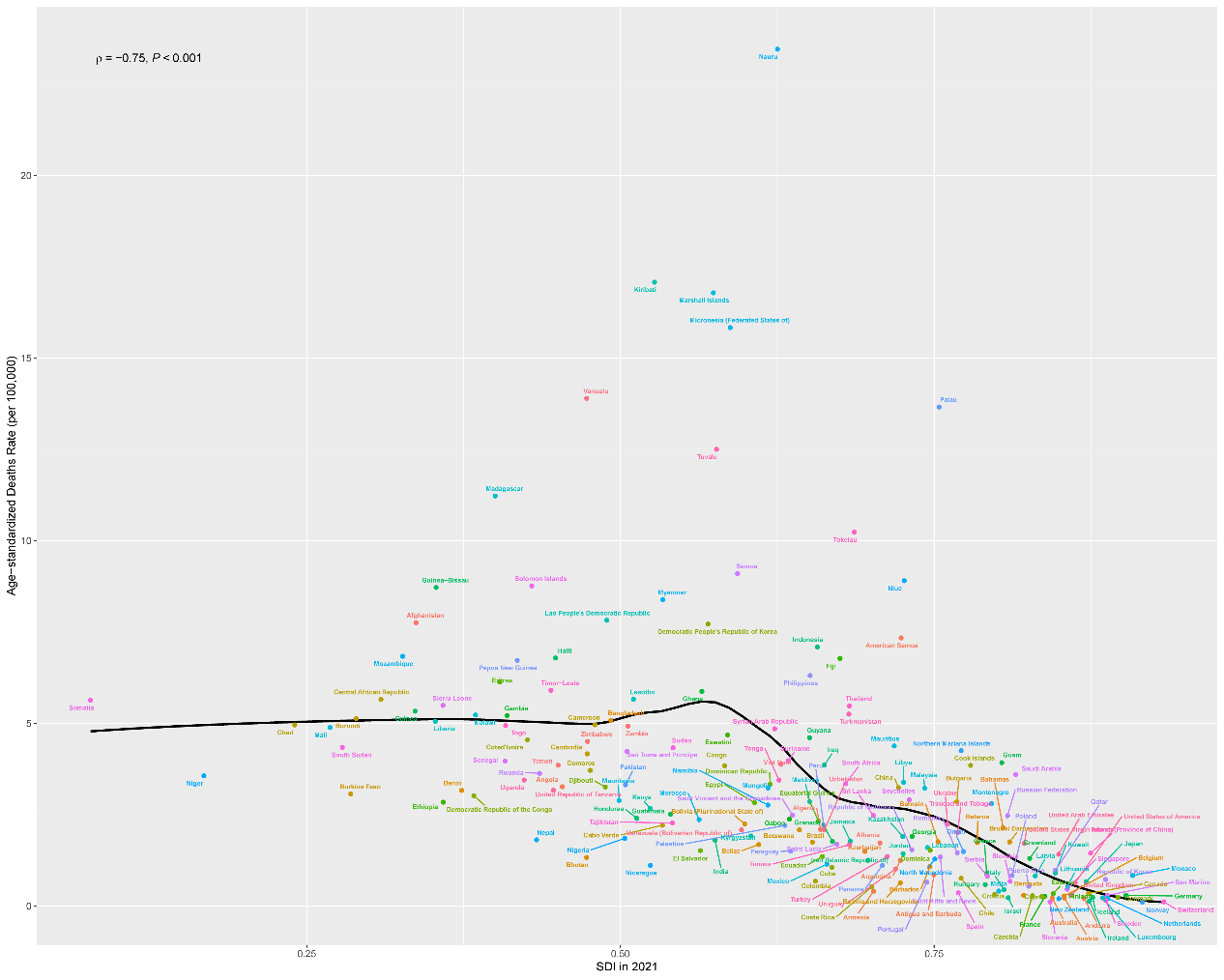


Supplementary Figure 5. Death rate of intracerebral hemorrhage by 204 countries and territories and Socio-demographic Index in 2021.
