# Supplementary material for: Global, regional, and national burden of intracerebral hemorrhage in adolescents and young adults and its predictions: a systematic analysis of the Global Burden of Disease Study 2021
Source: Front Neurol. 2025 Sep 8;16:1554952. doi: 10.3389/fneur.2025.1554952 (PMC12450654; doi:10.3389/fneur.2025.1554952)
Supplement: Supplementary file 2 [file Data_Sheet_2.pdf]

Supplementary Table 1 The age-standardized incidence rate and AAPC of intracerebral hemorrhage in adolescents and young adults globally, by SDI categories and GBD regions from 1990 to 2021

Category	Both sexes				Men				Women			
	Rates in 1990 (95% CI)	Rates in 2021 (95% CI)	AAPC,% (95% CI)	*P*	Rates in 1990 (95% CI)	Rates in 2021 (95% CI)	AAPC,% (95% CI)	*P*	Rates in 1990 (95% CI)	Rates in 2021 (95% CI)	AAPC,% (95% CI)	*P*
Global	11.85 (7.96 to 16.69)	8.14 (5.65 to 11.31)	-1.23 (-1.29 to -1.17)	<0.001	12.86 (8.66 to 18.19)	9.81 (6.88 to 13.53)	-0.91 (-0.96 to -0.86)	<0.001	10.81 (7.11 to 15.61)	6.44 (4.25 to 9.27)	-1.69 (-1.76 to -1.61)	<0.001
High SDI	6.3 (4.09 to 9.36)	4.02 (2.47 to 6.16)	-1.45 (-1.51 to -1.4)	<0.001	7.13 (4.68 to 10.46)	4.65 (2.96 to 6.96)	-1.38 (-1.47 to -1.29)	<0.001	5.44 (3.32 to 8.39)	3.34 (1.9 to 5.42)	-1.56 (-1.65 to -1.47)	<0.001
High-middle SDI	12.54 (8.35 to 17.75)	7.79 (5.27 to 11.05)	-1.57 (-1.67 to -1.47)	<0.001	14.44 (9.71 to 20.46)	9.96 (6.84 to 13.96)	-1.24 (-1.29 to -1.19)	<0.001	10.57 (6.83 to 15.51)	5.5 (3.43 to 8.27)	-2.13 (-2.22 to -2.03)	<0.001
Middle SDI	13.28 (8.78 to 18.98)	8.54 (5.88 to 11.88)	-1.45 (-1.48 to -1.41)	<0.001	14.17 (9.32 to 20.34)	10.55 (7.38 to 14.64)	-0.97 (-1.07 to -0.86)	<0.001	12.36 (8.03 to 18)	6.49 (4.2 to 9.48)	-2.09 (-2.18 to -2.01)	<0.001
Low-middle SDI	12.53 (8.6 to 17.39)	9.15 (6.41 to 12.58)	-1.04 (-1.1 to -0.99)	<0.001	12.97 (8.93 to 18.15)	10.54 (7.38 to 14.48)	-0.7 (-0.8 to -0.61)	<0.001	12.08 (8.13 to 17.15)	7.75 (5.22 to 10.9)	-1.46 (-1.52 to -1.4)	<0.001
Low SDI	14.92 (10.58 to 20.27)	9.68 (7.04 to 12.94)	-1.42 (-1.48 to -1.35)	<0.001	15.83 (11.19 to 21.9)	11.32 (8.22 to 15.22)	-1.1 (-1.15 to -1.06)	<0.001	14.03 (9.85 to 19.37)	8.1 (5.72 to 11.09)	-1.79 (-1.84 to -1.74)	<0.001
Andean Latin America	11.78 (8.83 to 15.54)	5.88 (4.13 to 8.08)	-2.29 (-2.39 to -2.18)	<0.001	12.48 (9.29 to 16.37)	6.76 (4.85 to 9.25)	-2.02 (-2.07 to -1.98)	<0.001	11.12 (8.05 to 15.1)	5 (3.22 to 7.3)	-2.62 (-2.76 to -2.48)	<0.001
Australasia	3.52 (2.12 to 5.36)	2.1 (1.07 to 3.61)	-1.64 (-1.73 to -1.56)	<0.001	3.88 (2.35 to 5.86)	2.29 (1.17 to 3.92)	-1.67 (-1.73 to -1.62)	<0.001	3.17 (1.79 to 5.12)	1.91 (0.93 to 3.34)	-1.61 (-1.72 to -1.49)	<0.001
Caribbean	10.93 (8.25 to 14.31)	8.35 (6.31 to 10.9)	-0.86 (-0.92 to -0.8)	<0.001	10.08 (7.44 to 13.41)	7.59 (5.55 to 10.19)	-0.92 (-1 to -0.84)	<0.001	11.74 (8.66 to 15.65)	9.12 (6.8 to 12.05)	-0.82 (-0.88 to -0.76)	<0.001
Central Asia	14.26 (11.01 to 18.48)	9.31 (7.06 to 12.19)	-1.39 (-1.55 to -1.23)	<0.001	15.8 (12.14 to 20.55)	10.89 (8.39 to 14.12)	-1.22 (-1.41 to -1.02)	<0.001	12.78 (9.46 to 16.93)	7.74 (5.49 to 10.63)	-1.63 (-1.72 to -1.54)	<0.001
Central Europe	9.08 (6.57 to 12.28)	4.15 (2.7 to 6.12)	-2.48 (-2.59 to -2.38)	<0.001	10.62 (7.8 to 14.18)	4.52 (3.05 to 6.43)	-2.75 (-2.82 to -2.68)	<0.001	7.5 (5.09 to 10.66)	3.77 (2.24 to 5.94)	-2.19 (-2.25 to -2.14)	<0.001
Central Latin America	8.02 (5.53 to 11.13)	4.47 (2.93 to 6.55)	-1.87 (-1.91 to -1.83)	<0.001	7.25 (5.01 to 10.07)	4.45 (2.96 to 6.45)	-1.57 (-1.61 to -1.53)	<0.001	8.75 (5.86 to 12.46)	4.5 (2.81 to 6.79)	-2.12 (-2.17 to -2.07)	<0.001
Central Sub-Saharan Africa	14.64 (10.67 to 19.76)	10.15 (7.53 to 13.53)	-1.19 (-1.23 to -1.15)	<0.001	17.39 (12.32 to 23.96)	12.45 (9.21 to 16.76)	-1.08 (-1.16 to -0.99)	<0.001	11.93 (8.46 to 16.57)	7.87 (5.6 to 10.88)	-1.36 (-1.38 to -1.33)	<0.001
East Asia	14.31 (9.15 to 21.11)	9.57 (6.47 to 13.53)	-1.33 (-1.4 to -1.25)	<0.001	15.83 (10.08 to 23.33)	12.96 (8.88 to 18.16)	-0.69 (-0.85 to -0.54)	<0.001	12.68 (7.95 to 18.9)	5.93 (3.73 to 8.9)	-2.47 (-2.64 to -2.3)	<0.001
Eastern Europe	8.25 (5.2 to 12.2)	7.76 (5.3 to 10.94)	-0.14 (-0.36 to 0.07)	0.19	10.33 (6.58 to 15.14)	10.19 (7.03 to 14.2)	-0.03 (-0.15 to 0.08)	0.549	6.19 (3.7 to 9.48)	5.32 (3.35 to 7.89)	-0.46 (-0.66 to -0.25)	<0.001
Eastern Sub-Saharan Africa	19.93 (14.51 to 26.61)	10.69 (7.9 to 14.06)	-2.01 (-2.06 to -1.96)	<0.001	22.72 (16.41 to 30.71)	13.8 (10.21 to 18.23)	-1.63 (-1.7 to -1.57)	<0.001	17.36 (12.37 to 23.65)	7.75 (5.48 to 10.55)	-2.59 (-2.67 to -2.52)	<0.001
High-income Asia Pacific	10.29 (6.87 to 14.66)	4.98 (2.93 to 7.96)	-2.33 (-2.47 to -2.19)	<0.001	11.59 (7.78 to 16.44)	5.66 (3.46 to 8.77)	-2.3 (-2.43 to -2.17)	<0.001	8.96 (5.78 to 13.3)	4.26 (2.26 to 7.25)	-2.37 (-2.47 to -2.27)	<0.001
High-income North America	4 (2.21 to 6.51)	3.15 (1.79 to 5)	-0.76 (-0.95 to -0.57)	<0.001	4.37 (2.49 to 7.05)	3.47 (2.02 to 5.45)	-0.75 (-0.89 to -0.61)	<0.001	3.64 (1.93 to 6.08)	2.83 (1.54 to 4.67)	-0.79 (-0.9 to -0.69)	<0.001
North Africa and Middle East	12.33 (9.02 to 16.4)	7.01 (5.05 to 9.48)	-1.8 (-1.88 to -1.72)	<0.001	12.51 (9.14 to 16.84)	7.53 (5.45 to 10.19)	-1.61 (-1.69 to -1.52)	<0.001	12.12 (8.67 to 16.49)	6.43 (4.48 to 8.94)	-2.01 (-2.1 to -1.91)	<0.001
Oceania	13.69 (10.08 to 18.22)	10.24 (7.73 to 13.47)	-0.94 (-0.98 to -0.91)	<0.001	15.88 (11.6 to 21.21)	12.17 (9.09 to 16)	-0.88 (-0.91 to -0.84)	<0.001	11.39 (7.97 to 15.69)	8.32 (5.91 to 11.49)	-1 (-1.05 to -0.96)	<0.001

South Asia	10.11<br>(6.51 to 14.72)	7.8<br>(5.16 to 11.2)	-0.88<br>(-0.96 to -0.8)	<0.001	10.13<br>(6.54 to 14.85)	8.85<br>(5.83 to 12.68)	-0.47<br>(-0.49 to -0.44)	<0.001	10.07<br>(6.32 to 15.06)	6.73<br>(4.3 to 9.9)	-1.32<br>(-1.36 to -1.27)	<0.001
Southeast Asia	18.25<br>(12.72 to 25.1)	13.49<br>(9.81 to 18.11)	-1.01<br>(-1.04 to -0.97)	<0.001	19.98<br>(14.08 to 27.55)	16.27<br>(11.94 to 21.72)	-0.66<br>(-0.77 to -0.55)	<0.001	16.56<br>(11.11 to 23.45)	10.64<br>(7.22 to 14.84)	-1.45<br>(-1.5 to -1.4)	<0.001
Southern Latin America	14.02<br>(10.18 to 18.74)	6.67<br>(4.41 to 9.67)	-2.37<br>(-2.44 to -2.29)	<0.001	15.58<br>(11.38 to 20.9)	7.2<br>(4.82 to 10.35)	-2.46<br>(-2.55 to -2.36)	<0.001	12.51<br>(8.65 to 17.26)	6.15<br>(3.88 to 9.11)	-2.27<br>(-2.35 to -2.2)	<0.001
Southern Sub-Saharan Africa	15.12<br>(10.07 to 21.61)	7.22<br>(5.02 to 10.03)	-2.41<br>(-2.55 to -2.28)	<0.001	17.29<br>(11.34 to 24.75)	9.11<br>(6.43 to 12.54)	-2.09<br>(-2.18 to -2)	<0.001	13.08<br>(8.52 to 19.06)	5.34<br>(3.48 to 7.73)	-2.9<br>(-3.03 to -2.77)	<0.001
Tropical Latin America	14.82<br>(9.4 to 21.88)	4.95<br>(3.16 to 7.24)	-3.53<br>(-3.6 to -3.46)	<0.001	15.89<br>(9.98 to 23.61)	5.31<br>(3.41 to 7.75)	-3.52<br>(-3.59 to -3.45)	<0.001	13.79<br>(8.7 to 20.59)	4.6<br>(2.84 to 6.94)	-3.54<br>(-3.64 to -3.44)	<0.001
Western Europe	5.01<br>(3.23 to 7.44)	2.61<br>(1.4 to 4.4)	-2.09<br>(-2.14 to -2.04)	<0.001	5.91<br>(3.94 to 8.56)	2.88<br>(1.61 to 4.7)	-2.3<br>(-2.36 to -2.24)	<0.001	4.08<br>(2.42 to 6.42)	2.33<br>(1.16 to 4.06)	-1.8<br>(-1.84 to -1.76)	<0.001
Western Sub-Saharan Africa	13.85<br>(9.69 to 18.98)	10.17<br>(7.34 to 13.75)	-1.01<br>(-1.06 to -0.95)	<0.001	14.82<br>(10.22 to 20.87)	11.49<br>(8.24 to 15.59)	-0.8<br>(-0.91 to -0.7)	<0.001	12.96<br>(9.09 to 17.82)	8.98<br>(6.37 to 12.29)	-1.19<br>(-1.27 to -1.12)	<0.001

Supplementary Table 2 Age-specific incidence rate and AAPC of intracerebral hemorrhage burden from 1990 to 2021 globally and by SDI categories

location	sex	15 to 19			
		Rates in 1990 (95% CI)	Rates in 2021 (95% CI)	AAPC,% (95% CI)	*P*
Global	Female	5.13 (2.83 to 8.36)	3.61 (2 to 5.96)	-1.13 (-1.16 to -1.1)	<0.001
	Male	5.08 (3 to 8.17)	3.99 (2.37 to 6.41)	-0.79 (-0.85 to -0.73)	<0.001
High SDI	Female	3.44 (1.66 to 6.13)	2.39 (1.07 to 4.35)	-1.15 (-1.28 to -1.02)	<0.001
	Male	3.29 (1.69 to 5.8)	2.13 (0.94 to 3.93)	-1.38 (-1.48 to -1.28)	<0.001
High-middle SDI	Female	5.33 (2.93 to 8.74)	3.61 (1.89 to 6.14)	-1.23 (-1.35 to -1.12)	<0.001
	Male	5.65 (3.26 to 9.06)	3.56 (1.97 to 5.94)	-1.48 (-1.61 to -1.35)	<0.001
Middle SDI	Female	5.51 (3.03 to 9.06)	3.77 (2.05 to 6.33)	-1.21 (-1.24 to -1.18)	<0.001
	Male	5.25 (3.08 to 8.42)	4.03 (2.37 to 6.52)	-0.84 (-0.96 to -0.71)	<0.001
Low-middle SDI	Female	5.23 (2.93 to 8.48)	3.84 (2.12 to 6.16)	-0.98 (-1.06 to -0.89)	<0.001
	Male	4.44 (2.56 to 7.3)	3.85 (2.24 to 6.2)	-0.46 (-0.52 to -0.4)	<0.001
Low SDI	Female	5.21 (3.02 to 8.34)	3.61 (2.05 to 5.73)	-1.17 (-1.21 to -1.14)	<0.001
	Male	7.26 (4.79 to 10.81)	5.35 (3.48 to 7.86)	-1.01 (-1.08 to -0.95)	<0.001

location	sex	20 to 24			
		Rates in 1990 (95% CI)	Rates in 2021 (95% CI)	AAPC,% (95% CI)	*P*
Global	Female	6.69 (4.39 to 10.16)	4.34 (2.8 to 6.57)	-1.4 (-1.47 to -1.34)	<0.001
	Male	6.95 (4.73 to 10.05)	5.35 (3.74 to 7.58)	-0.86 (-0.92 to -0.8)	<0.001
High SDI	Female	3.94 (2.25 to 6.59)	2.66 (1.35 to 4.68)	-1.25 (-1.34 to -1.15)	<0.001
	Male	4.23 (2.61 to 6.73)	2.71 (1.51 to 4.62)	-1.43 (-1.51 to -1.35)	<0.001
High-middle SDI	Female	6.78 (4.35 to 10.35)	4.12 (2.47 to 6.65)	-1.62 (-1.69 to -1.55)	<0.001
	Male	7.79 (5.25 to 11.24)	5.02 (3.36 to 7.31)	-1.41 (-1.55 to -1.27)	<0.001
Middle SDI	Female	7.29 (4.74 to 11.08)	4.46 (2.85 to 6.88)	-1.59 (-1.65 to -1.53)	<0.001
	Male	7.27 (4.93 to 10.65)	5.52 (3.82 to 7.81)	-0.89 (-1.01 to -0.77)	<0.001
Low-middle SDI	Female	7.07 (4.78 to 10.51)	4.78 (3.16 to 7.11)	-1.28 (-1.35 to -1.22)	<0.001
	Male	6.44 (4.42 to 9.25)	5.4 (3.78 to 7.68)	-0.58 (-0.63 to -0.53)	<0.001
Low SDI	Female	7.37 (5.2 to 10.56)	4.59 (3.13 to 6.73)	-1.54 (-1.59 to -1.49)	<0.001
	Male	9.44 (6.9 to 12.92)	6.97 (5.2 to 9.37)	-1 (-1.06 to -0.94)	<0.001

location	sex	25 to 29			
		Rates in 1990 (95% CI)	Rates in 2021 (95% CI)	AAPC,% (95% CI)	*P*
Global	Female	8.84 (5.85 to 13.43)	5.18 (3.44 to 7.67)	-1.75 (-1.88 to -1.62)	<0.001
	Male	9.51 (6.08 to 14.1)	7.18 (4.78 to 10.31)	-0.95 (-1.02 to -0.88)	<0.001
High SDI	Female	4.88 (3.02 to 7.71)	3.07 (1.71 to 5.27)	-1.47 (-1.58 to -1.37)	<0.001
	Male	5.59 (3.47 to 8.58)	3.66 (2.15 to 5.84)	-1.37 (-1.41 to -1.32)	<0.001
High-middle SDI	Female	8.74 (5.77 to 13.47)	4.57 (2.81 to 7.09)	-2.06 (-2.33 to -1.78)	<0.001
	Male	10.48 (6.68 to 15.47)	7.06 (4.47 to 10.28)	-1.31 (-1.51 to -1.1)	<0.001
Middle SDI	Female	9.96 (6.52 to 15.24)	5.24 (3.46 to 7.87)	-2.08 (-2.21 to -1.95)	<0.001
	Male	10.21 (6.37 to 15.35)	7.58 (5.01 to 10.88)	-0.97 (-1.12 to -0.83)	<0.001
Low-middle SDI	Female	9.6 (6.48 to 14.28)	5.92 (4 to 8.56)	-1.58 (-1.67 to -1.5)	<0.001
	Male	9.61 (6.34 to 13.94)	7.62 (5.1 to 10.92)	-0.78 (-0.82 to -0.75)	<0.001
Low SDI	Female	10.44 (7.16 to 15.09)	5.9 (4.08 to 8.11)	-1.86 (-1.93 to -1.79)	<0.001
	Male	11.88 (8.04 to 16.78)	8.67 (6.11 to 11.94)	-1.02 (-1.08 to -0.96)	<0.001

location	sex	30 to 34			
		Rates in 1990 (95% CI)	Rates in 2021 (95% CI)	AAPC,% (95% CI)	*P*
Global	Female	13.73 (9.76 to 18.32)	7.82 (5.45 to 10.39)	-1.83 (-1.91 to -1.75)	<0.001
	Male	16.75 (12.29 to 22.34)	12.81 (9.62 to 16.76)	-0.88 (-0.92 to -0.84)	<0.001
High SDI	Female	6.63 (4.35 to 9.38)	3.83 (2.36 to 5.72)	-1.76 (-1.83 to -1.69)	<0.001
	Male	9.19 (6.63 to 12.68)	5.96 (4.22 to 8.39)	-1.41 (-1.51 to -1.31)	<0.001
High-middle SDI	Female	13.18 (9.07 to 17.8)	6.37 (4.24 to 8.81)	-2.34 (-2.48 to -2.19)	<0.001
	Male	18.66 (13.67 to 25.06)	13.36 (9.95 to 17.8)	-1.1 (-1.2 to -1)	<0.001
Middle SDI	Female	15.89 (11.15 to 21.2)	7.79 (5.34 to 10.4)	-2.3 (-2.41 to -2.2)	<0.001
	Male	18.61 (13.41 to 25.04)	13.95 (10.41 to 18.28)	-0.95 (-0.99 to -0.91)	<0.001
Low-middle SDI	Female	15.68 (11.41 to 20.74)	9.64 (6.84 to 12.64)	-1.6 (-1.67 to -1.52)	<0.001
	Male	17.5 (13.17 to 23)	13.9 (10.55 to 17.95)	-0.78 (-0.87 to -0.7)	<0.001
Low SDI	Female	18.47 (14.06 to 23.8)	10.28 (7.78 to 13.21)	-1.91 (-1.96 to -1.86)	<0.001
	Male	20.03 (15.16 to 26.61)	14.17 (10.99 to 18.24)	-1.13 (-1.18 to -1.08)	<0.001

location	sex	35 to 39			
		Rates in 1990 (95% CI)	Rates in 2021 (95% CI)	AAPC,% (95% CI)	*P*
Global	Female	21.47 (13.98 to 30.14)	12.22 (8.26 to 16.98)	-1.84 (-1.9 to -1.78)	<0.001
	Male	28.64 (19.01 to 39.74)	21.66 (15.32 to 29.1)	-0.94 (-1.03 to -0.84)	<0.001
High SDI	Female	8.92 (5.81 to 12.89)	5.07 (3.23 to 7.39)	-1.81 (-1.9 to -1.73)	<0.001
	Male	14.63 (9.89 to 20.1)	9.6 (6.61 to 13.03)	-1.38 (-1.47 to -1.29)	<0.001
High-middle SDI	Female	20.52 (13.19 to 29.41)	9.46 (6.2 to 13.46)	-2.54 (-2.66 to -2.42)	<0.001
	Male	32.6 (21.78 to 45.43)	22.93 (16.02 to 31.27)	-1.18 (-1.28 to -1.08)	<0.001
Middle SDI	Female	25.32 (16.17 to 36.39)	12.11 (7.98 to 17.05)	-2.41 (-2.5 to -2.31)	<0.001
	Male	32.5 (20.79 to 46.4)	23.85 (16.93 to 32.6)	-1.02 (-1.18 to -0.86)	<0.001
Low-middle SDI	Female	25.03 (16.56 to 34.6)	15.9 (10.94 to 21.74)	-1.5 (-1.59 to -1.41)	<0.001
	Male	29.63 (20.15 to 40.96)	24.15 (16.88 to 32.52)	-0.7 (-0.79 to -0.61)	<0.001
Low SDI	Female	31.56 (21.91 to 42.83)	17.68 (12.74 to 23.6)	-1.9 (-1.98 to -1.82)	<0.001
	Male	33.43 (23.13 to 46.26)	23.45 (16.81 to 31.25)	-1.16 (-1.22 to -1.1)	<0.001

Supplementary Table 3 The age-standardized rates and AAPC of intracerebral hemorrhage in adolescents and young adults by different countries, regions and territories from 1990 to 2021

Category	Age-standardized incidence rate				Age-standardized DALYs rate				Age-standardized Deaths rate			
	Rates in 1990 (95% CI)	Rates in 2021 (95% CI)	AAPC,% (95% CI)	*P*	Rates in 1990 (95% CI)	Rates in 2021 (95% CI)	AAPC,% (95% CI)	*P*	Rates in 1990 (95% CI)	Rates in 2021 (95% CI)	AAPC,% (95% CI)	*P*
Afghanistan	27.87 (20.97 to 36.66)	15.11 (11.68 to 19.58)	-2.02 (-2.11 to -1.93)	<0.001	748.55 (466.82 to 1090.71)	477.59 (301.66 to 714.48)	-1.35 (-1.6 to -1.09)	<0.001	12.2 (7.45 to 17.97)	7.76 (4.81 to 11.76)	-1.36 (-1.66 to -1.07)	<0.001
Angola	15.91 (11.73 to 21.23)	9.55 (6.87 to 13.03)	-1.68 (-1.77 to -1.6)	<0.001	334.27 (222.57 to 475.96)	204.61 (133.54 to 295.71)	-1.52 (-2.2 to -0.83)	<0.001	5.37 (3.49 to 7.75)	3.26 (2.07 to 4.81)	-1.54 (-2.26 to -0.81)	<0.001
Algeria	14.79 (10.8 to 19.85)	6.87 (4.83 to 9.64)	-2.46 (-2.58 to -2.33)	<0.001	323.53 (220.76 to 450.99)	137.17 (91.34 to 201.28)	-2.66 (-3 to -2.32)	<0.001	5.1 (3.39 to 7.25)	2.1 (1.33 to 3.19)	-2.8 (-3.06 to -2.55)	<0.001
Azerbaijan	13.5 (9.81 to 18.13)	7.14 (5 to 10.04)	-2.03 (-2.29 to -1.78)	<0.001	268.52 (190.35 to 361.82)	101.17 (66.68 to 140.48)	-3.12 (-4.24 to -2)	<0.001	4.25 (2.92 to 5.85)	1.49 (0.93 to 2.15)	-3.34 (-4.54 to -2.13)	<0.001
Armenia	6.64 (4.24 to 9.96)	4.52 (2.62 to 7.29)	-1.21 (-1.35 to -1.08)	<0.001	111.28 (97.38 to 126.61)	34.39 (28.43 to 41.42)	-3.71 (-4.82 to -2.59)	<0.001	1.69 (1.46 to 1.93)	0.4 (0.34 to 0.47)	-4.54 (-5.91 to -3.15)	<0.001
Albania	10.64 (7.84 to 14.19)	7.37 (5.29 to 9.89)	-1.22 (-1.32 to -1.12)	<0.001	243.08 (187.52 to 302.06)	119.42 (85.45 to 161.7)	-2.56 (-3.58 to -1.54)	<0.001	3.64 (2.75 to 4.61)	1.72 (1.16 to 2.43)	-2.72 (-3.83 to -1.6)	<0.001
Argentina	16.56 (12.2 to 21.82)	7 (4.71 to 9.9)	-2.74 (-2.89 to -2.6)	<0.001	294.46 (257.49 to 335.28)	88.96 (76.36 to 103.26)	-3.86 (-4.79 to -2.92)	<0.001	4.62 (4 to 5.27)	1.25 (1.06 to 1.46)	-4.24 (-5.31 to -3.15)	<0.001
American Samoa	19.55 (14.57 to 25.97)	15.82 (11.79 to 20.72)	-0.68 (-0.75 to -0.61)	<0.001	515.43 (372.65 to 701.73)	456.68 (317.83 to 627.88)	-0.46 (-0.78 to -0.14)	0.006	8.27 (5.88 to 11.49)	7.34 (4.95 to 10.25)	-0.45 (-0.77 to -0.13)	0.006
Antigua and Barbuda	8.45 (5.95 to 11.81)	5.05 (3.28 to 7.48)	-1.65 (-1.71 to -1.59)	<0.001	210.66 (182.43 to 243.92)	58.01 (48.22 to 69.37)	-4.21 (-6.32 to -2.06)	<0.001	3.5 (3 to 4.06)	0.85 (0.7 to 1.03)	-4.59 (-6.83 to -2.3)	<0.001
Australia	3.66 (2.24 to 5.53)	2.11 (1.09 to 3.6)	-1.73 (-1.82 to -1.64)	<0.001	39.26 (33.23 to 45.89)	18.67 (15.38 to 22.27)	-2.23 (-2.94 to -1.52)	<0.001	0.53 (0.44 to 0.63)	0.21 (0.17 to 0.26)	-2.76 (-3.42 to -2.09)	<0.001
Austria	5.1 (3.2 to 7.81)	2.98 (1.5 to 5.16)	-1.72 (-1.89 to -1.55)	<0.001	72.85 (62.68 to 84.34)	20.93 (17.49 to 24.9)	-3.89 (-4.42 to -3.35)	<0.001	1.07 (0.92 to 1.25)	0.22 (0.18 to 0.26)	-4.9 (-5.68 to -4.12)	<0.001
Andorra	3.06 (1.6 to 5.24)	2.48 (1.25 to 4.36)	-0.66 (-0.76 to -0.55)	<0.001	37.19 (25.44 to 53.16)	19.48 (13.51 to 26.46)	-2.15 (-2.51 to -1.78)	<0.001	0.51 (0.32 to 0.79)	0.21 (0.13 to 0.33)	-2.87 (-3.38 to -2.36)	<0.001
Burkina Faso	11.05 (7.64 to 15.52)	9.43 (6.76 to 12.93)	-0.52 (-0.57 to -0.47)	<0.001	238.9 (163.39 to 336.29)	194.85 (127.24 to 278.24)	-0.65 (-0.91 to -0.39)	<0.001	3.81 (2.52 to 5.5)	3.06 (1.91 to 4.45)	-0.7 (-0.97 to -0.42)	<0.001
Burundi	28.94 (22.19 to 37.46)	12.7 (9.59 to 16.69)	-2.67 (-2.76 to -2.58)	<0.001	733.54 (490.17 to 1054.56)	318.45 (207.5 to 454.5)	-2.7 (-2.86 to -2.55)	<0.001	11.86 (7.78 to 17.23)	5.13 (3.26 to 7.41)	-2.71 (-2.87 to -2.55)	<0.001
Benin	13.42 (9.46 to 18.68)	10.57 (7.47 to 14.58)	-0.82 (-0.89 to -0.74)	<0.001	262.15 (183.25 to 364.15)	203.8 (136.59 to 284.18)	-0.83 (-0.99 to -0.67)	<0.001	4.12 (2.78 to 5.85)	3.16 (2.04 to 4.52)	-0.87 (-1.04 to -0.7)	<0.001
Bhutan	8.1 (5.3 to 11.76)	4.77 (2.93 to 7.23)	-1.69 (-1.73 to -1.65)	<0.001	163.28 (89.99 to 258.86)	86.07 (48.12 to 136.03)	-2.08 (-2.28 to -1.88)	<0.001	2.63 (1.4 to 4.28)	1.33 (0.69 to 2.19)	-2.21 (-2.43 to -2)	<0.001
Bangladesh	16.26 (11.84 to 21.71)	10.91 (7.83 to 14.89)	-1.31 (-1.34 to -1.27)	<0.001	534.45 (342.63 to 742.04)	310.42 (187.99 to 453.25)	-1.55 (-1.98 to -1.12)	<0.001	8.85 (5.61 to 12.38)	5.08 (2.99 to 7.5)	-1.59 (-2.02 to -1.16)	<0.001
Bolivia (Plurinational State of)	12.74 (9.09 to 17.28)	5.18 (3.48 to 7.37)	-2.91 (-2.97 to -2.85)	<0.001	427.21 (278.04 to 622.38)	142.44 (85.33 to 215.13)	-3.53 (-3.67 to -3.39)	<0.001	6.9 (4.41 to 10.18)	2.25 (1.3 to 3.44)	-3.6 (-3.73 to -3.47)	<0.001
Belize	6.9 (4.8 to 9.59)	5.79 (4.11 to 7.98)	-0.58 (-0.65 to -0.51)	<0.001	174.32 (144.61 to 207.33)	104.12 (85.51 to 123.94)	-1.28 (-2.08 to -0.47)	0.002	2.78 (2.28 to 3.32)	1.68 (1.37 to 2.02)	-1.19 (-2.06 to -0.31)	0.008
Botswana	12.9 (9.28 to 17.49)	9.17 (6.66 to 12.36)	-1.14 (-1.2 to -1.07)	<0.001	308.68 (159.04 to 492.16)	134.05 (69.93 to 209.25)	-2.66 (-3.66 to -1.64)	<0.001	5.02 (2.49 to 8.14)	2.08 (1 to 3.36)	-3.01 (-4.19 to -1.81)	<0.001
Brazil	14.95 (9.45 to 22.12)	4.95 (3.15 to 7.25)	-3.56 (-3.63 to -3.49)	<0.001	337.9 (320.72 to 357.1)	107.33 (100.76 to 113.89)	-3.59 (-4.03 to -3.14)	<0.001	5.74 (5.43 to 6.06)	1.75 (1.64 to 1.86)	-3.72 (-4.18 to -3.26)	<0.001

Bosnia and Herzegovina	5.05 (3.22 to 7.65)	3.59 (2.15 to 5.65)	-1.08 (-1.29 to -0.87)	<0.001	111.04 (78.95 to 149.51)	49.26 (34.67 to 65.62)	-2.73 (-3.55 to -1.9)	<0.001	1.67 (1.15 to 2.31)	0.63 (0.4 to 0.9)	-3.24 (-4.31 to -2.16)	<0.001
Barbados	7.68 (5.43 to 10.84)	4.92 (3.24 to 7.2)	-1.41 (-1.52 to -1.3)	<0.001	147.9 (126.53 to 172.71)	69.94 (54.41 to 91.26)	-2.27 (-3.12 to -1.42)	<0.001	2.41 (2.05 to 2.83)	1.08 (0.81 to 1.44)	-2.29 (-3.42 to -1.15)	<0.001
Bahrain	9.86 (6.74 to 13.97)	5.8 (3.64 to 8.74)	-1.68 (-1.99 to -1.36)	<0.001	214.59 (163.72 to 277.87)	122.06 (89.79 to 163.34)	-1.76 (-2.46 to -1.07)	<0.001	3.1 (2.28 to 4.1)	1.76 (1.23 to 2.43)	-1.74 (-2.37 to -1.11)	<0.001
Bulgaria	15.94 (12.83 to 19.92)	7.9 (5.99 to 10.39)	-2.28 (-2.34 to -2.22)	<0.001	385.66 (335.75 to 436.24)	182.56 (149.99 to 216.58)	-2.3 (-2.92 to -1.68)	<0.001	6.18 (5.36 to 7)	2.85 (2.33 to 3.43)	-2.36 (-3.02 to -1.7)	<0.001
Belarus	9.64 (6.95 to 13.19)	6.87 (4.8 to 9.63)	-1.14 (-1.37 to -0.91)	<0.001	157.3 (129.29 to 191.17)	108.08 (85.83 to 134.2)	-1.53 (-2.46 to -0.59)	0.001	2.54 (2.06 to 3.12)	1.73 (1.35 to 2.19)	-1.56 (-2.56 to -0.56)	0.002
Bahamas	10.66 (8.12 to 13.95)	7.43 (5.58 to 9.82)	-1.17 (-1.23 to -1.11)	<0.001	242.62 (204.76 to 283.17)	130.03 (98.64 to 165.63)	-2.07 (-2.88 to -1.24)	<0.001	4.05 (3.39 to 4.74)	2.14 (1.6 to 2.77)	-2.11 (-2.96 to -1.25)	<0.001
Brunei Darussalam	14.44 (10 to 20.23)	5.75 (3.69 to 8.67)	-2.94 (-3 to -2.87)	<0.001	263.29 (188.33 to 361.69)	113.53 (83.3 to 148.79)	-2.61 (-3.19 to -2.04)	<0.001	4.08 (2.86 to 5.72)	1.75 (1.23 to 2.38)	-2.57 (-3.23 to -1.91)	<0.001
Bermuda	5.91 (3.56 to 9.19)	4.06 (2.18 to 6.82)	-1.14 (-1.22 to -1.05)	<0.001	66.19 (54.28 to 79.71)	24.34 (19.58 to 30.56)	-3.46 (-3.79 to -3.12)	<0.001	0.99 (0.79 to 1.21)	0.29 (0.22 to 0.39)	-4.14 (-4.59 to -3.69)	<0.001
Belgium	5.32 (3.49 to 7.82)	2.3 (1.18 to 3.96)	-2.67 (-2.87 to -2.47)	<0.001	112.36 (98.6 to 127.79)	22.96 (19.39 to 26.9)	-5.19 (-5.9 to -4.47)	<0.001	1.76 (1.53 to 2.01)	0.27 (0.23 to 0.32)	-6.09 (-7.16 to -5.01)	<0.001
Chad	14.94 (10.77 to 20.11)	14.18 (10.64 to 18.61)	-0.18 (-0.22 to -0.13)	<0.001	326.58 (222.83 to 460.39)	315.03 (208.72 to 469.64)	-0.11 (-0.43 to 0.2)	0.481	5.17 (3.44 to 7.4)	4.96 (3.18 to 7.56)	-0.13 (-0.47 to 0.2)	0.44
Central African Republic	17.57 (12.95 to 23.3)	14.02 (10.85 to 18.13)	-0.74 (-0.85 to -0.63)	<0.001	417.1 (259.56 to 624.37)	346.43 (196.7 to 548.55)	-0.66 (-1.07 to -0.25)	0.002	6.76 (4.11 to 10.3)	5.66 (3.1 to 9.11)	-0.64 (-1.05 to -0.22)	0.003
Coted'Ivoire	16.88 (12.23 to 22.79)	12.31 (9.09 to 16.31)	-1.04 (-1.12 to -0.97)	<0.001	366.03 (246.39 to 517.51)	290.85 (185.98 to 424.33)	-0.75 (-1.08 to -0.41)	<0.001	5.73 (3.76 to 8.28)	4.55 (2.75 to 6.79)	-0.75 (-1 to -0.5)	<0.001
Cambodia	15.25 (10.85 to 20.68)	12.71 (9.61 to 16.69)	-0.58 (-0.7 to -0.47)	<0.001	487.38 (353.25 to 669.71)	261.6 (177.87 to 390.69)	-1.98 (-2.11 to -1.84)	<0.001	8.04 (5.76 to 11.19)	4.16 (2.74 to 6.38)	-2.08 (-2.22 to -1.95)	<0.001
Comoros	19.82 (14.25 to 26.63)	10.07 (7.07 to 13.81)	-2.18 (-2.34 to -2.02)	<0.001	479.38 (205.62 to 719.01)	232.84 (154.42 to 334.8)	-3.08 (-5.54 to -0.55)	0.017	7.74 (3.17 to 11.73)	3.71 (2.4 to 5.4)	-3.11 (-5.61 to -0.55)	0.018
Cameroon	15.65 (11.39 to 21.09)	13.66 (10.2 to 18.12)	-0.46 (-0.73 to -0.2)	0.001	383.64 (254.01 to 533.57)	312.81 (191.95 to 460.95)	-0.67 (-0.83 to -0.51)	<0.001	6.15 (3.95 to 8.7)	4.96 (2.91 to 7.5)	-0.7 (-0.87 to -0.54)	<0.001
Cabo Verde	17.43 (12.34 to 23.93)	9.45 (6.54 to 13.1)	-2.04 (-2.12 to -1.95)	<0.001	403.62 (293.89 to 535.42)	151.27 (100.06 to 212.6)	-3.11 (-3.63 to -2.59)	<0.001	6.43 (4.56 to 8.65)	2.21 (1.35 to 3.25)	-3.38 (-3.99 to -2.76)	<0.001
Congo	19.63 (14.69 to 25.8)	9.37 (6.79 to 12.72)	-2.41 (-2.48 to -2.35)	<0.001	402.44 (253.48 to 603.5)	237.41 (146.64 to 372.13)	-1.54 (-2.04 to -1.04)	<0.001	6.56 (4.01 to 10.02)	3.84 (2.27 to 6.19)	-1.55 (-2.07 to -1.03)	<0.001
Colombia	7.57 (5.26 to 10.59)	3.39 (1.98 to 5.43)	-2.57 (-2.65 to -2.5)	<0.001	156.43 (136.6 to 177.66)	45.9 (36.81 to 55.95)	-4 (-4.93 to -3.07)	<0.001	2.5 (2.17 to 2.85)	0.68 (0.53 to 0.84)	-4.2 (-5.27 to -3.12)	<0.001
Cuba	8.62 (6.12 to 11.96)	4.53 (2.87 to 6.86)	-2.05 (-2.16 to -1.93)	<0.001	158 (137.77 to 179.55)	67.8 (56.25 to 81.39)	-2.56 (-4 to -1.09)	0.001	2.59 (2.24 to 2.95)	1.06 (0.87 to 1.28)	-2.67 (-4.25 to -1.07)	0.001
Costa Rica	6.17 (3.84 to 9.39)	4.12 (2.43 to 6.57)	-1.26 (-1.32 to -1.2)	<0.001	73.44 (62.2 to 85.74)	38.01 (31.44 to 45.58)	-2.18 (-2.75 to -1.62)	<0.001	1.1 (0.92 to 1.3)	0.52 (0.42 to 0.64)	-2.44 (-3.12 to -1.76)	<0.001
China	14.24 (9.03 to 21.14)	9.44 (6.33 to 13.41)	-1.35 (-1.43 to -1.28)	<0.001	316.69 (265.83 to 385.04)	208.12 (172.46 to 247.04)	-1.26 (-1.53 to -1)	<0.001	4.96 (4.11 to 6.09)	3.24 (2.63 to 3.89)	-1.27 (-1.56 to -0.97)	<0.001
Chile	9.14 (5.8 to 13.51)	6.1 (3.68 to 9.41)	-1.28 (-1.33 to -1.23)	<0.001	142.16 (123.35 to 161.57)	60.95 (51.45 to 71.45)	-2.74 (-3.32 to -2.16)	<0.001	2.09 (1.8 to 2.38)	0.76 (0.64 to 0.89)	-3.27 (-3.99 to -2.55)	<0.001
Cook Islands	18 (13.2 to 23.92)	13.15 (9.65 to 17.92)	-1.01 (-1.08 to -0.93)	<0.001	450.91 (309.85 to 658.66)	251.79 (167.57 to 365.94)	-1.86 (-2.01 to -1.71)	<0.001	7.14 (4.76 to 10.73)	3.85 (2.42 to 5.81)	-1.97 (-2.14 to -1.81)	<0.001
Croatia	7.4 (5.34 to 10.31)	2.96 (1.68 to 4.79)	-2.93 (-2.97 to -2.89)	<0.001	140.2 (120.46 to 161.26)	30.01 (24.16 to 37.04)	-4.87 (-5.52 to -4.22)	<0.001	2.13 (1.83 to 2.46)	0.32 (0.26 to 0.4)	-5.9 (-6.78 to -5.02)	<0.001
Czechia	6.42 (4.11 to 9.44)	3.53 (1.89 to 5.93)	-1.88 (-1.93 to -1.82)	<0.001	95 (80.84 to 111.4)	28.36 (22.31 to 35.54)	-3.8 (-4.36 to -3.24)	<0.001	1.37 (1.15 to 1.6)	0.28 (0.22 to 0.35)	-5.12 (-6.17 to -4.06)	<0.001

Cyprus	4.54 (2.63 to 7.33)	3.04 (1.61 to 5.12)	-1.29 (-1.33 to -1.25)	<0.001	61.25 (42.38 to 84.05)	23.34 (16.86 to 31.02)	-3.07 (-3.93 to -2.22)	<0.001	0.86 (0.55 to 1.25)	0.26 (0.16 to 0.37)	-3.84 (-5.02 to -2.64)	<0.001
Canada	3.79 (2.04 to 6.37)	3.01 (1.68 to 4.84)	-0.74 (-0.8 to -0.68)	<0.001	40.83 (34.36 to 48.02)	29.23 (24.28 to 34.82)	-0.98 (-1.52 to -0.44)	<0.001	0.51 (0.43 to 0.6)	0.34 (0.28 to 0.41)	-1.04 (-1.78 to -0.3)	0.006
Democratic Republic of the Congo	13.72 (9.77 to 18.83)	10.29 (7.62 to 13.7)	-0.93 (-0.99 to -0.86)	<0.001	261.26 (163.23 to 392.23)	189.16 (114.91 to 293.95)	-1.07 (-1.21 to -0.92)	<0.001	4.13 (2.48 to 6.34)	3.02 (1.77 to 4.8)	-1.04 (-1.18 to -0.89)	<0.001
Djibouti	16.28 (11.65 to 22.3)	9.75 (6.89 to 13.52)	-1.68 (-1.76 to -1.61)	<0.001	341.45 (215.95 to 507.67)	204.43 (122.17 to 324.57)	-1.51 (-1.9 to -1.12)	<0.001	5.5 (3.36 to 8.3)	3.26 (1.86 to 5.28)	-1.54 (-1.93 to -1.14)	<0.001
Dominican Republic	10.56 (7.88 to 14.02)	9.29 (6.92 to 12.26)	-0.4 (-0.42 to -0.37)	<0.001	271.44 (202.86 to 355.84)	204.15 (141.48 to 293.57)	-1.12 (-2.66 to 0.45)	0.16	4.48 (3.31 to 5.93)	3.33 (2.28 to 4.84)	-1.16 (-2.73 to 0.44)	0.154
Dominica	7.74 (5.31 to 11.08)	4.8 (3.18 to 7.05)	-1.55 (-1.61 to -1.49)	<0.001	117.44 (86.33 to 155.88)	95.03 (66.74 to 131.59)	-0.78 (-1.13 to -0.43)	<0.001	1.87 (1.35 to 2.51)	1.52 (1.04 to 2.15)	-0.75 (-1.12 to -0.37)	<0.001
Democratic People's Republic of Korea	18.71 (13.63 to 24.89)	16.52 (12.51 to 21.47)	-0.42 (-0.43 to -0.4)	<0.001	430.88 (283.35 to 641.27)	475.14 (305.07 to 748.75)	0.33 (0.25 to 0.41)	<0.001	6.78 (4.28 to 10.33)	7.72 (4.8 to 12.44)	0.44 (0.35 to 0.53)	<0.001
Denmark	3.78 (2.41 to 5.73)	2.59 (1.28 to 4.59)	-1.18 (-1.44 to -0.92)	<0.001	72.01 (62.01 to 83.45)	21.31 (17.7 to 25.54)	-3.83 (-5.17 to -2.48)	<0.001	1.08 (0.91 to 1.26)	0.24 (0.19 to 0.29)	-4.67 (-6.4 to -2.92)	<0.001
Ethiopia	23.87 (15.54 to 34.52)	7.54 (4.99 to 10.78)	-3.7 (-3.86 to -3.55)	<0.001	478.65 (348.56 to 684.97)	179.1 (129.84 to 230.5)	-3.16 (-3.37 to -2.94)	<0.001	7.71 (5.55 to 11.18)	2.84 (2.02 to 3.7)	-3.2 (-3.42 to -2.98)	<0.001
Eritrea	23.16 (17.16 to 30.57)	14.96 (11.17 to 19.84)	-1.43 (-1.52 to -1.34)	<0.001	648.66 (427.66 to 939.44)	374.96 (224.59 to 586.1)	-1.85 (-2.16 to -1.54)	<0.001	10.66 (6.94 to 15.55)	6.13 (3.59 to 9.74)	-1.86 (-2.17 to -1.55)	<0.001
El Salvador	12.68 (9.71 to 16.46)	4.42 (2.95 to 6.41)	-3.33 (-3.46 to -3.21)	<0.001	258.01 (196.02 to 336.5)	96.1 (65.55 to 134.4)	-3.09 (-5.06 to -1.08)	0.003	4.1 (3.07 to 5.38)	1.52 (1 to 2.17)	-3.43 (-4.94 to -1.9)	<0.001
Eswatini	8.88 (6.32 to 12.1)	9.53 (7.15 to 12.57)	0.2 (0.09 to 0.32)	0.001	246.78 (146.54 to 362.65)	286.69 (159.84 to 454.49)	0.48 (0.24 to 0.73)	<0.001	4 (2.31 to 5.94)	4.68 (2.52 to 7.54)	0.51 (0.25 to 0.78)	<0.001
Egypt	9.65 (6.79 to 13.21)	5.74 (3.95 to 8.06)	-1.66 (-1.75 to -1.56)	<0.001	465.05 (318.34 to 634.12)	182.56 (118.6 to 259.61)	-2.93 (-3.41 to -2.44)	<0.001	7.46 (5.03 to 10.24)	2.83 (1.79 to 4.1)	-3.12 (-3.61 to -2.62)	<0.001
Equatorial Guinea	17.14 (12.47 to 22.75)	6.28 (4.22 to 9.01)	-3.18 (-3.27 to -3.09)	<0.001	396.83 (251.99 to 615.85)	148 (79.76 to 247.53)	-3.03 (-3.69 to -2.36)	<0.001	6.45 (4.02 to 10.18)	2.32 (1.13 to 3.97)	-3.13 (-3.85 to -2.4)	<0.001
Ecuador	11.3 (8.42 to 14.8)	5.69 (3.97 to 7.94)	-2.26 (-2.33 to -2.18)	<0.001	308.44 (265.78 to 352.08)	87.67 (67.96 to 110.7)	-4.08 (-4.94 to -3.21)	<0.001	5 (4.3 to 5.73)	1.35 (1.02 to 1.74)	-4.24 (-5.13 to -3.34)	<0.001
Estonia	8.13 (5.8 to 11.28)	3.44 (1.83 to 5.86)	-2.67 (-2.82 to -2.53)	<0.001	95.86 (77.15 to 117.27)	27.06 (21.77 to 33.59)	-3.86 (-5.2 to -2.49)	<0.001	1.48 (1.16 to 1.84)	0.33 (0.26 to 0.42)	-4.7 (-6.27 to -3.1)	<0.001
Fiji	19.79 (14.76 to 26)	13.45 (10.01 to 17.71)	-1.26 (-1.3 to -1.22)	<0.001	617.08 (465.48 to 806.16)	429.31 (303.94 to 578.9)	-1.08 (-1.32 to -0.85)	<0.001	9.82 (7.28 to 12.96)	6.77 (4.68 to 9.29)	-1.11 (-1.39 to -0.83)	<0.001
France	4.17 (2.76 to 5.98)	2.37 (1.27 to 3.89)	-1.83 (-1.92 to -1.73)	<0.001	75.63 (65.98 to 86.99)	21.9 (18.19 to 25.88)	-3.9 (-4.75 to -3.05)	<0.001	1.17 (1.01 to 1.36)	0.26 (0.21 to 0.32)	-4.65 (-5.76 to -3.53)	<0.001
Finland	4.36 (2.87 to 6.4)	3.59 (2.11 to 5.58)	-0.68 (-0.81 to -0.55)	<0.001	90.83 (76.79 to 106.24)	26 (21.68 to 30.84)	-3.88 (-5.11 to -2.63)	<0.001	1.4 (1.17 to 1.65)	0.3 (0.24 to 0.36)	-4.74 (-5.52 to -3.95)	<0.001
Guinea	16.5 (12.22 to 21.94)	15.28 (11.45 to 20.02)	-0.26 (-0.35 to -0.17)	<0.001	376.3 (265.53 to 524.87)	338.83 (218.21 to 489.6)	-0.32 (-0.6 to -0.05)	0.022	5.96 (4.13 to 8.46)	5.33 (3.32 to 7.83)	-0.35 (-0.63 to -0.06)	0.018
Guinea-Bissau	24.22 (18.37 to 31.42)	17.47 (13.49 to 22.49)	-1.09 (-1.16 to -1.01)	<0.001	779.15 (519.7 to 1125.04)	536.26 (359.14 to 780.9)	-1.19 (-1.32 to -1.05)	<0.001	12.67 (8.31 to 18.5)	8.72 (5.74 to 12.87)	-1.18 (-1.3 to -1.07)	<0.001
Gambia	14.02 (9.66 to 19.52)	11.77 (8.47 to 16.05)	-0.55 (-0.78 to -0.31)	<0.001	369.88 (251.42 to 531.8)	327.8 (214.22 to 484.69)	-0.43 (-1.41 to 0.57)	0.399	5.87 (3.86 to 8.58)	5.21 (3.34 to 7.82)	-0.42 (-1.45 to 0.62)	0.431
Guatemala	17.98 (14.71 to 21.98)	8.71 (6.8 to 11.12)	-2.3 (-2.6 to -2.01)	<0.001	335.17 (291.81 to 387.95)	156.46 (127.21 to 190.1)	-2.15 (-3.32 to -0.96)	<0.001	5.4 (4.67 to 6.29)	2.51 (2.01 to 3.08)	-2.14 (-3.37 to -0.91)	0.001
Ghana	22.74 (16.97 to 30.4)	16.32 (12.16 to 21.55)	-1.12 (-1.19 to -1.05)	<0.001	624.97 (435.55 to 848.09)	367.94 (252.45 to 509.19)	-1.71 (-1.88 to -1.54)	<0.001	10.12 (6.91 to 13.89)	5.87 (3.93 to 8.27)	-1.76 (-1.94 to -1.57)	<0.001
Gabon	15 (10.7 to 20.64)	8.28 (5.88 to 11.45)	-1.93 (-1.97 to -1.88)	<0.001	266.86 (176.96 to 384.94)	151.34 (88.35 to 236.42)	-1.81 (-2.12 to -1.49)	<0.001	4.26 (2.71 to 6.31)	2.37 (1.3 to 3.83)	-1.86 (-2.19 to -1.53)	<0.001

Guyana	19.42<br>(15.53 to 24.03)	11.04<br>(8.72 to 13.86)	-1.85<br>(-1.98 to -1.72)	<0.001	501.33<br>(408.35 to 597.52)	276.93<br>(204.93 to 370.79)	-1.78<br>(-3 to -0.56)	0.004	8.35<br>(6.78 to 9.97)	4.61<br>(3.38 to 6.23)	-1.78<br>(-3.1 to -0.44)	0.01
Grenada	12.68<br>(9.65 to 16.57)	6.03<br>(4.33 to 8.28)	-2.39<br>(-2.48 to -2.29)	<0.001	359.45<br>(301.54 to 425.77)	107.89<br>(84.98 to 132.81)	-4.03<br>(-4.65 to -3.42)	<0.001	6.02<br>(5.03 to 7.15)	1.77<br>(1.38 to 2.2)	-4.09<br>(-4.73 to -3.45)	<0.001
Georgia	26.47<br>(20.61 to 33.86)	11.56<br>(8.61 to 15.29)	-2.73<br>(-3.08 to -2.38)	<0.001	369.01<br>(306.24 to 438.52)	128.87<br>(103.4 to 156.15)	-3.17<br>(-5.83 to -0.44)	0.023	5.63<br>(4.58 to 6.78)	1.9<br>(1.5 to 2.36)	-3.89<br>(-6.65 to -1.06)	0.007
Greece	9.06<br>(6.35 to 12.42)	4.52<br>(2.88 to 6.72)	-2.29<br>(-2.41 to -2.16)	<0.001	174.8<br>(153.26 to 198.05)	62.25<br>(53.06 to 71.72)	-3.4<br>(-4.17 to -2.63)	<0.001	2.69<br>(2.35 to 3.06)	0.89<br>(0.75 to 1.04)	-3.55<br>(-4.35 to -2.74)	<0.001
Guam	11.33<br>(8.14 to 15.5)	12.56<br>(9.4 to 16.51)	0.33<br>(0.23 to 0.42)	<0.001	225.83<br>(162.67 to 296.94)	252.91<br>(193.48 to 328.62)	0.38<br>(-0.88 to 1.66)	0.556	3.35<br>(2.29 to 4.52)	3.92<br>(2.94 to 5.2)	0.73<br>(-0.59 to 2.07)	0.28
Greenland	10.13<br>(7.1 to 13.9)	4.71<br>(3.14 to 6.83)	-2.51<br>(-2.64 to -2.39)	<0.001	249.86<br>(170.52 to 362.93)	85.76<br>(56.63 to 117.68)	-3.4<br>(-3.6 to -3.2)	<0.001	4.01<br>(2.64 to 5.9)	1.3<br>(0.8 to 1.85)	-3.53<br>(-3.74 to -3.32)	<0.001
Germany	5.02<br>(3.19 to 7.7)	2.67<br>(1.37 to 4.62)	-2.01<br>(-2.11 to -1.9)	<0.001	94.56<br>(81.09 to 110.3)	24.43<br>(20.55 to 28.7)	-4.31<br>(-5.2 to -3.41)	<0.001	1.44<br>(1.22 to 1.7)	0.29<br>(0.24 to 0.35)	-5.02<br>(-6.08 to -3.96)	<0.001
Haiti	19.5<br>(14.89 to 25.38)	12.25<br>(9.43 to 15.78)	-1.51<br>(-1.57 to -1.45)	<0.001	692.16<br>(460.13 to 985.94)	407.94<br>(258.18 to 618.25)	-1.52<br>(-1.99 to -1.06)	<0.001	11.59<br>(7.68 to 16.52)	6.79<br>(4.25 to 10.34)	-1.55<br>(-2.01 to -1.08)	<0.001
Honduras	8.31<br>(5.84 to 11.48)	3.72<br>(2.26 to 5.73)	-2.59<br>(-2.69 to -2.5)	<0.001	408.01<br>(290.39 to 552.84)	146.52<br>(72.81 to 237.09)	-3.35<br>(-3.78 to -2.92)	<0.001	6.76<br>(4.74 to 9.21)	2.4<br>(1.14 to 3.95)	-3.39<br>(-3.84 to -2.94)	<0.001
Hungary	11.37<br>(8.62 to 15.02)	3.85<br>(2.24 to 6.19)	-3.47<br>(-3.57 to -3.36)	<0.001	219.74<br>(190.11 to 254.99)	46.86<br>(37.7 to 57.56)	-4.93<br>(-6.6 to -3.23)	<0.001	3.52<br>(3.02 to 4.11)	0.59<br>(0.47 to 0.72)	-5.69<br>(-7.7 to -3.64)	<0.001
India	9.15<br>(5.67 to 13.65)	7<br>(4.53 to 10.22)	-0.9<br>(-0.99 to -0.82)	<0.001	185.45<br>(143.14 to 229.92)	114.11<br>(92.85 to 136.76)	-1.58<br>(-2.16 to -0.99)	<0.001	2.97<br>(2.27 to 3.71)	1.81<br>(1.45 to 2.2)	-1.63<br>(-2.27 to -0.98)	<0.001
Indonesia	24.54<br>(15.95 to 35.63)	14.52<br>(9.9 to 20.19)	-1.72<br>(-1.76 to -1.68)	<0.001	662.52<br>(563.15 to 766.01)	436.44<br>(351.9 to 577.49)	-1.33<br>(-1.42 to -1.24)	<0.001	10.79<br>(9.13 to 12.53)	7.09<br>(5.64 to 9.51)	-1.34<br>(-1.44 to -1.24)	<0.001
Iraq	17.7<br>(13.48 to 22.96)	12.98<br>(9.92 to 16.78)	-1.03<br>(-1.07 to -0.99)	<0.001	519.99<br>(377.53 to 696.11)	251.97<br>(174.72 to 365.74)	-2.38<br>(-2.63 to -2.12)	<0.001	8.27<br>(5.92 to 11.21)	3.86<br>(2.59 to 5.81)	-2.52<br>(-2.77 to -2.27)	<0.001
Iran (Islamic Republic of)	6.51<br>(4.01 to 9.8)	4.11<br>(2.6 to 6.08)	-1.48<br>(-1.53 to -1.44)	<0.001	142.39<br>(119.22 to 162.82)	86.27<br>(76.97 to 96.33)	-1.59<br>(-1.69 to -1.48)	<0.001	2.14<br>(1.78 to 2.47)	1.25<br>(1.11 to 1.4)	-1.7<br>(-1.82 to -1.57)	<0.001
Italy	5.61<br>(3.21 to 8.95)	2.76<br>(1.53 to 4.57)	-2.28<br>(-2.3 to -2.26)	<0.001	97.3<br>(91.45 to 103.62)	33.69<br>(30.26 to 37.37)	-3.58<br>(-4.34 to -2.81)	<0.001	1.46<br>(1.39 to 1.52)	0.45<br>(0.41 to 0.48)	-4<br>(-5.25 to -2.73)	<0.001
Israel	3.54<br>(2.04 to 5.64)	2.01<br>(0.96 to 3.63)	-1.81<br>(-1.91 to -1.71)	<0.001	78.79<br>(67.79 to 91.05)	19.36<br>(15.91 to 23.09)	-4.37<br>(-5.22 to -3.5)	<0.001	1.2<br>(1.02 to 1.4)	0.22<br>(0.18 to 0.27)	-5.1<br>(-6.19 to -4)	<0.001
Ireland	4.46<br>(2.59 to 7.17)	2.78<br>(1.39 to 4.89)	-1.5<br>(-1.63 to -1.37)	<0.001	54.49<br>(46.54 to 63)	15.41<br>(12.23 to 19.11)	-4.05<br>(-4.33 to -3.77)	<0.001	0.76<br>(0.65 to 0.89)	0.13<br>(0.1 to 0.16)	-5.37<br>(-5.77 to -4.96)	<0.001
Iceland	4.47<br>(2.58 to 7.17)	2.77<br>(1.37 to 4.9)	-1.52<br>(-1.77 to -1.26)	<0.001	62.69<br>(53.22 to 73.1)	21.19<br>(17.39 to 25.66)	-3.37<br>(-3.8 to -2.95)	<0.001	0.89<br>(0.74 to 1.06)	0.23<br>(0.19 to 0.29)	-4.23<br>(-5.17 to -3.29)	<0.001
Jamaica	7.68<br>(5.35 to 10.71)	6.26<br>(4.46 to 8.68)	-0.64<br>(-0.72 to -0.56)	<0.001	173.1<br>(139.45 to 214.93)	109.79<br>(80.84 to 149.08)	-1.29<br>(-2.6 to 0.04)	0.057	2.78<br>(2.22 to 3.48)	1.78<br>(1.27 to 2.46)	-1.21<br>(-2.63 to 0.23)	0.1
Jordan	8.1<br>(5.43 to 11.69)	4.45<br>(2.67 to 7.03)	-1.89<br>(-2.11 to -1.67)	<0.001	301.32<br>(226.69 to 388.12)	98.74<br>(72.75 to 130.29)	-3.47<br>(-3.97 to -2.97)	<0.001	4.7<br>(3.49 to 6.13)	1.43<br>(1.01 to 1.93)	-3.7<br>(-4.25 to -3.16)	<0.001
Japan	6.15<br>(3.31 to 10.08)	4.57<br>(2.61 to 7.44)	-0.94<br>(-1.13 to -0.76)	<0.001	86.78<br>(80.44 to 94.24)	52.41<br>(47.11 to 58.56)	-1.55<br>(-1.99 to -1.11)	<0.001	1.24<br>(1.18 to 1.3)	0.67<br>(0.64 to 0.7)	-1.94<br>(-2.79 to -1.08)	<0.001
Kenya	12.43<br>(7.87 to 18.28)	8.84<br>(5.91 to 12.46)	-1.06<br>(-1.21 to -0.91)	<0.001	208.64<br>(156.53 to 262.28)	169.58<br>(122.91 to 228.8)	-0.7<br>(-0.8 to -0.6)	<0.001	3.25<br>(2.36 to 4.16)	2.67<br>(1.88 to 3.66)	-0.66<br>(-0.76 to -0.56)	<0.001
Kiribati	67.48<br>(53.61 to 83.91)	55.94<br>(45.78 to 67.52)	-0.64<br>(-0.72 to -0.57)	<0.001	1055.79<br>(752.74 to 1426.02)	1057.31<br>(706.65 to 1513.19)	-0.01<br>(-0.06 to 0.05)	0.792	16.87<br>(11.7 to 23.09)	17.08<br>(11.15 to 24.88)	0.03<br>(-0.03 to 0.08)	0.306
Kyrgyzstan	18.27<br>(14.31 to 23.28)	7.47<br>(5.58 to 10.08)	-2.9<br>(-2.95 to -2.85)	<0.001	330.33<br>(279.56 to 383.49)	120.79<br>(97.25 to 149.49)	-3.23<br>(-4.27 to -2.17)	<0.001	5.31<br>(4.45 to 6.21)	1.92<br>(1.52 to 2.39)	-3.28<br>(-4.48 to -2.06)	<0.001
Kazakhstan	13.93<br>(10.59 to 18.29)	9.17<br>(6.8 to 12.22)	-1.3<br>(-1.76 to -0.84)	<0.001	224.92<br>(197.13 to 254.25)	123.95<br>(93.2 to 153.71)	-1.95<br>(-2.96 to -0.93)	<0.001	3.45<br>(3.02 to 3.92)	1.9<br>(1.37 to 2.41)	-1.94<br>(-3.03 to -0.83)	0.001

Kuwait	6.71 (4.2 to 9.96)	5.37 (3.41 to 8.06)	-0.7 (-0.78 to -0.62)	<0.001	122.21 (102.6 to 142.96)	68.56 (54.82 to 85.26)	-1.81 (-2.9 to -0.71)	0.001	1.72 (1.43 to 2.05)	0.91 (0.7 to 1.15)	-2.01 (-3.23 to -0.77)	0.002
Liberia	16.4 (11.93 to 22.02)	11.64 (8.43 to 15.64)	-1.13 (-1.16 to -1.1)	<0.001	368.21 (257.15 to 521.09)	318.06 (197.73 to 479.72)	-0.53 (-0.98 to -0.07)	0.023	5.78 (3.93 to 8.3)	5.05 (3.07 to 7.74)	-0.49 (-0.96 to -0.02)	0.041
Lao People's Democratic Republic	20.67 (15.15 to 27.4)	15.58 (12.02 to 20.21)	-0.93 (-1.01 to -0.84)	<0.001	813.68 (565.4 to 1148.7)	481.81 (323.87 to 705.85)	-1.68 (-1.82 to -1.54)	<0.001	13.53 (9.29 to 19.16)	7.82 (5.11 to 11.58)	-1.76 (-1.84 to -1.67)	<0.001
Lesotho	6.77 (4.64 to 9.56)	11.2 (8.6 to 14.46)	1.63 (1.51 to 1.75)	<0.001	162.32 (85.48 to 252.19)	342.03 (211.71 to 513.25)	2.51 (2.03 to 2.99)	<0.001	2.6 (1.29 to 4.13)	5.66 (3.41 to 8.56)	2.62 (2.1 to 3.13)	<0.001
Libya	9.81 (7 to 13.32)	7.1 (5.07 to 9.63)	-1.06 (-1.11 to -1.02)	<0.001	281.58 (192.8 to 393.87)	219.11 (141.54 to 322.1)	-0.71 (-1.42 to 0.01)	0.053	4.32 (2.86 to 6.13)	3.38 (2.11 to 5.07)	-0.68 (-1.46 to 0.11)	0.09
Lebanon	11.09 (7.89 to 15.18)	6.32 (4.24 to 8.97)	-1.8 (-1.87 to -1.74)	<0.001	371.51 (252.96 to 525.27)	111.89 (81.77 to 148.87)	-3.77 (-4.11 to -3.42)	<0.001	5.98 (3.98 to 8.59)	1.6 (1.12 to 2.21)	-4.14 (-4.55 to -3.74)	<0.001
Latvia	7.21 (5.15 to 10.15)	3.24 (1.93 to 5.14)	-2.56 (-2.64 to -2.49)	<0.001	134.57 (112.94 to 159.58)	54.95 (42.38 to 69.36)	-2.69 (-4.48 to -0.86)	0.004	2.14 (1.77 to 2.55)	0.82 (0.61 to 1.07)	-3.09 (-5.45 to -0.66)	0.013
Lithuania	6.41 (4.41 to 9.1)	3.96 (2.61 to 5.86)	-1.49 (-1.76 to -1.22)	<0.001	93.42 (76.25 to 112)	37.27 (30.55 to 45.79)	-3.08 (-4.83 to -1.3)	0.001	1.47 (1.18 to 1.78)	0.53 (0.43 to 0.66)	-3.42 (-5.41 to -1.39)	0.001
Luxembourg	6.26 (4.24 to 8.77)	2.34 (1.23 to 3.94)	-3.12 (-3.23 to -3.02)	<0.001	132.93 (113.56 to 154.96)	20.29 (16.62 to 24.37)	-5.97 (-6.73 to -5.21)	<0.001	2.06 (1.75 to 2.41)	0.22 (0.18 to 0.27)	-7.02 (-8.01 to -6.01)	<0.001
Mali	15.33 (11.14 to 20.73)	10.26 (7.42 to 14.02)	-1.31 (-1.37 to -1.25)	<0.001	462.09 (307.23 to 657.1)	308.58 (205.82 to 437.12)	-1.29 (-1.57 to -1.02)	<0.001	7.44 (4.85 to 10.72)	4.88 (3.17 to 7.03)	-1.35 (-1.63 to -1.06)	<0.001
Mozambique	13.45 (9.78 to 18.35)	14.77 (11.22 to 19.2)	0.3 (0.24 to 0.35)	<0.001	307.96 (210.47 to 425.63)	413.39 (252.28 to 625.25)	0.97 (0.79 to 1.14)	<0.001	5 (3.37 to 7.01)	6.83 (4.07 to 10.47)	1.03 (0.81 to 1.25)	<0.001
Malawi	14.45 (10.77 to 19.07)	9.84 (7.47 to 12.92)	-1.25 (-1.31 to -1.19)	<0.001	364.09 (251.48 to 507.91)	321.75 (210.31 to 455.68)	-0.38 (-0.66 to -0.09)	0.009	5.84 (3.96 to 8.24)	5.23 (3.37 to 7.5)	-0.32 (-0.6 to -0.04)	0.026
Madagascar	37.86 (29.72 to 47.69)	22.36 (17.15 to 28.8)	-1.71 (-1.79 to -1.63)	<0.001	997.44 (750.57 to 1285.28)	688.46 (447.31 to 974.07)	-1.19 (-1.3 to -1.07)	<0.001	16.24 (12.15 to 21.06)	11.22 (7.18 to 16.05)	-1.18 (-1.3 to -1.06)	<0.001
Mauritania	17.09 (12.03 to 23.64)	8.88 (6.16 to 12.47)	-2.13 (-2.21 to -2.06)	<0.001	409.46 (281.65 to 586.21)	187.26 (115.02 to 297.82)	-2.6 (-2.72 to -2.48)	<0.001	6.49 (4.35 to 9.49)	2.89 (1.66 to 4.75)	-2.67 (-2.8 to -2.54)	<0.001
Myanmar	21.14 (16.02 to 28)	15.01 (11.74 to 19.2)	-1.12 (-1.25 to -0.99)	<0.001	929.1 (653.68 to 1284.43)	514.64 (358.04 to 716.35)	-1.92 (-2.11 to -1.72)	<0.001	15.44 (10.76 to 21.48)	8.38 (5.75 to 11.74)	-1.98 (-2.18 to -1.78)	<0.001
Morocco	12.11 (8.7 to 16.37)	6.2 (4.21 to 8.66)	-2.19 (-2.29 to -2.1)	<0.001	368.88 (232.61 to 534.9)	153 (92.47 to 244.25)	-2.81 (-2.94 to -2.68)	<0.001	5.88 (3.63 to 8.68)	2.36 (1.33 to 3.9)	-2.91 (-3.08 to -2.74)	<0.001
Marshall Islands	31.75 (24.29 to 40.8)	33.99 (26.89 to 42.64)	0.2 (0.08 to 0.32)	0.001	975.9 (667.08 to 1367.18)	1013.25 (678.5 to 1464.86)	0.13 (0.04 to 0.22)	0.005	15.84 (10.68 to 22.33)	16.78 (11.06 to 24.53)	0.2 (0.12 to 0.27)	<0.001
Micronesia (Federated States of)	37.31 (28.03 to 49.21)	31.79 (24.2 to 41.46)	-0.57 (-0.62 to -0.52)	<0.001	1156.92 (785.47 to 1595.11)	960.56 (642.35 to 1375.84)	-0.61 (-0.64 to -0.58)	<0.001	19.25 (12.84 to 26.74)	15.83 (10.39 to 22.88)	-0.64 (-0.67 to -0.6)	<0.001
Mongolia	24.81 (19.05 to 32.29)	21.01 (16.43 to 26.52)	-0.58 (-0.78 to -0.38)	<0.001	357.93 (263.83 to 476.41)	210.01 (152.65 to 281.05)	-1.68 (-2.81 to -0.53)	0.004	5.62 (4.02 to 7.65)	3.23 (2.26 to 4.41)	-1.73 (-2.94 to -0.5)	0.006
Maldives	27.5 (20.4 to 36.27)	10.26 (7.33 to 14.01)	-3.2 (-3.37 to -3.03)	<0.001	778.74 (580.21 to 1083.44)	189.11 (135.58 to 254.24)	-4.54 (-5.07 to -4)	<0.001	12.74 (9.33 to 18.08)	2.85 (1.94 to 3.98)	-4.78 (-5.33 to -4.23)	<0.001
Mexico	6.88 (4.18 to 10.43)	4.15 (2.54 to 6.33)	-1.62 (-1.68 to -1.57)	<0.001	122.44 (116.57 to 128.68)	74.33 (66.81 to 82.5)	-1.49 (-2.22 to -0.76)	<0.001	1.89 (1.82 to 1.97)	1.14 (1.02 to 1.27)	-1.46 (-2.19 to -0.73)	<0.001
Mauritius	16.27 (12.11 to 21.42)	9.62 (7.01 to 12.96)	-1.7 (-1.79 to -1.6)	<0.001	354.43 (305.98 to 406.69)	270.98 (229.14 to 315.6)	-0.21 (-1.36 to 0.95)	0.717	5.75 (4.93 to 6.63)	4.38 (3.69 to 5.13)	-0.18 (-1.44 to 1.09)	0.779
Malaysia	18.65 (13.6 to 24.83)	12.28 (8.85 to 16.51)	-1.36 (-1.42 to -1.3)	<0.001	353.65 (274.3 to 452.9)	213.85 (163.25 to 278.15)	-1.62 (-1.94 to -1.3)	<0.001	5.38 (4.04 to 7)	3.22 (2.39 to 4.28)	-1.62 (-1.98 to -1.25)	<0.001
Montenegro	15.98 (12.31 to 20.58)	8.64 (6.43 to 11.41)	-2.03 (-2.22 to -1.84)	<0.001	382.27 (300.19 to 478.02)	183.09 (138.96 to 240.14)	-2.17 (-2.94 to -1.39)	<0.001	6.13 (4.73 to 7.73)	2.8 (2.08 to 3.76)	-2.33 (-3.44 to -1.2)	<0.001
Malta	5.78 (3.68 to 8.65)	3.54 (2.03 to 5.66)	-1.61 (-1.68 to -1.54)	<0.001	99.94 (86.63 to 114.01)	32.71 (27.51 to 38.58)	-3.45 (-3.98 to -2.92)	<0.001	1.47 (1.26 to 1.71)	0.41 (0.34 to 0.49)	-4.01 (-4.71 to -3.3)	<0.001

Monaco	4.47 (2.76 to 6.84)	3.24 (1.89 to 5.17)	-1.05 (-1.11 to -0.99)	<0.001	99.7 (68.25 to 138.65)	56.38 (34.59 to 85.53)	-1.82 (-2.03 to -1.61)	<0.001	1.56 (1.03 to 2.22)	0.83 (0.46 to 1.34)	-2 (-2.21 to -1.79)	<0.001
Niger	16.1 (11.58 to 21.94)	11.47 (8.37 to 15.42)	-1.1 (-1.15 to -1.05)	<0.001	314.41 (201.54 to 471.13)	228.72 (139.98 to 348.21)	-1 (-1.2 to -0.79)	<0.001	4.9 (3.03 to 7.51)	3.56 (2.08 to 5.56)	-1.02 (-1.34 to -0.7)	<0.001
Nepal	9.46 (6.31 to 13.66)	5.56 (3.54 to 8.26)	-1.7 (-1.77 to -1.64)	<0.001	212.97 (131.6 to 317.06)	114.68 (69.25 to 176.84)	-1.97 (-2.11 to -1.82)	<0.001	3.47 (2.07 to 5.25)	1.81 (1.04 to 2.87)	-2.07 (-2.22 to -1.91)	<0.001
Nigeria	10.96 (6.82 to 16.34)	7.23 (4.69 to 10.55)	-1.33 (-1.45 to -1.21)	<0.001	218.6 (168.85 to 285.81)	122.8 (88.21 to 167.09)	-1.88 (-2.06 to -1.71)	<0.001	3.44 (2.61 to 4.59)	1.85 (1.27 to 2.6)	-2.02 (-2.21 to -1.84)	<0.001
Nicaragua	6.71 (4.5 to 9.81)	3.63 (2.08 to 5.87)	-1.96 (-2.07 to -1.85)	<0.001	159.55 (121.78 to 204.59)	71.54 (52.25 to 96.77)	-2.69 (-3.28 to -2.11)	<0.001	2.58 (1.94 to 3.33)	1.11 (0.78 to 1.54)	-2.83 (-3.45 to -2.21)	<0.001
Namibia	10.46 (7.39 to 14.56)	7.05 (5.06 to 9.56)	-1.28 (-1.34 to -1.23)	<0.001	236.11 (130.96 to 348.2)	171.36 (91.22 to 285.98)	-1.01 (-1.34 to -0.67)	<0.001	3.8 (2.02 to 5.69)	2.76 (1.39 to 4.7)	-0.99 (-1.34 to -0.63)	<0.001
Nauru	45.8 (35.65 to 58.7)	40.82 (32.37 to 51.54)	-0.44 (-0.5 to -0.37)	<0.001	1442.95 (954.42 to 2155.21)	1427.1 (953.44 to 2153.71)	-0.05 (-0.17 to 0.07)	0.431	23.78 (15.44 to 35.94)	23.45 (15.45 to 35.68)	-0.05 (-0.18 to 0.08)	0.438
Niue	26.27 (19.58 to 34.65)	17.79 (13.18 to 23.57)	-1.28 (-1.31 to -1.24)	<0.001	604.99 (390.83 to 923.24)	568.04 (398.4 to 789.89)	-0.14 (-0.64 to 0.35)	0.569	9.68 (6.04 to 15.03)	8.9 (6.09 to 12.66)	-0.21 (-0.72 to 0.3)	0.422
North Macedonia	12.37 (9.29 to 16.32)	6.62 (4.65 to 9.1)	-2.02 (-2.14 to -1.91)	<0.001	265.27 (208.1 to 327.24)	92.15 (65.56 to 126.84)	-3.14 (-3.62 to -2.66)	<0.001	4.11 (3.14 to 5.15)	1.28 (0.86 to 1.83)	-3.76 (-4.48 to -3.03)	<0.001
Northern Mariana Islands	17.76 (12.74 to 24.28)	12.16 (8.9 to 16.51)	-1.18 (-1.52 to -0.83)	<0.001	458.81 (291.8 to 654.77)	270.54 (193.68 to 370.5)	-1.65 (-2.31 to -0.98)	<0.001	7.48 (4.55 to 10.91)	4.25 (2.96 to 5.92)	-1.75 (-2.53 to -0.96)	<0.001
New Zealand	2.86 (1.43 to 5.02)	2.01 (0.96 to 3.68)	-1.09 (-1.27 to -0.91)	<0.001	42.6 (37.39 to 48.41)	18.46 (15.36 to 22.13)	-2.72 (-3.13 to -2.3)	<0.001	0.6 (0.53 to 0.69)	0.2 (0.17 to 0.23)	-3.67 (-5.45 to -1.86)	<0.001
Netherlands	4.13 (2.39 to 6.69)	2.7 (1.36 to 4.75)	-1.37 (-1.43 to -1.31)	<0.001	55.62 (47.61 to 63.86)	19.54 (16.09 to 23.49)	-3.4 (-3.73 to -3.07)	<0.001	0.8 (0.67 to 0.92)	0.21 (0.17 to 0.24)	-4.39 (-5.28 to -3.49)	<0.001
Norway	3.26 (1.57 to 5.79)	2.16 (0.99 to 3.97)	-1.29 (-1.51 to -1.07)	<0.001	33.9 (30.76 to 37.44)	12.16 (10.02 to 14.77)	-3.39 (-3.73 to -3.05)	<0.001	0.46 (0.43 to 0.5)	0.1 (0.1 to 0.11)	-4.56 (-5.86 to -3.25)	<0.001
Oman	12.58 (9.23 to 16.75)	7.01 (5 to 9.65)	-1.93 (-2.05 to -1.8)	<0.001	252.64 (169.97 to 364.38)	105.55 (73.22 to 146.25)	-2.81 (-3.23 to -2.38)	<0.001	3.81 (2.45 to 5.63)	1.49 (0.98 to 2.16)	-3 (-3.47 to -2.53)	<0.001
Papua New Guinea	8.05 (5.4 to 11.61)	6.41 (4.46 to 9.07)	-0.72 (-0.79 to -0.65)	<0.001	492.3 (285.15 to 794.05)	405.79 (238.58 to 621.93)	-0.65 (-0.83 to -0.48)	<0.001	8.26 (4.7 to 13.42)	6.72 (3.84 to 10.42)	-0.69 (-0.87 to -0.51)	<0.001
Pakistan	12.79 (8.01 to 18.86)	11.03 (7.35 to 15.73)	-0.49 (-0.65 to -0.33)	<0.001	188.5 (125.19 to 252.8)	209.79 (144.96 to 290.58)	0.36 (0.16 to 0.57)	<0.001	2.98 (1.92 to 4.06)	3.32 (2.22 to 4.67)	0.36 (0.15 to 0.57)	0.001
Palestine	6.92 (4.62 to 9.96)	4.35 (2.74 to 6.49)	-1.46 (-1.59 to -1.33)	<0.001	311.19 (215.76 to 433.73)	145.65 (107.85 to 190.29)	-2.39 (-2.88 to -1.9)	<0.001	4.86 (3.28 to 6.88)	2.2 (1.59 to 2.93)	-2.48 (-2.99 to -1.97)	<0.001
Paraguay	9.51 (6.89 to 12.8)	4.96 (3.29 to 7.26)	-2.09 (-2.14 to -2.03)	<0.001	208.64 (159.6 to 267.76)	93.23 (65.58 to 127.81)	-2.35 (-3.48 to -1.21)	<0.001	3.45 (2.61 to 4.45)	1.5 (1.03 to 2.09)	-2.52 (-3.51 to -1.53)	<0.001
Philippines	6.93 (4.1 to 10.86)	14.75 (10.14 to 20.62)	2.48 (2.13 to 2.84)	<0.001	374.09 (335.16 to 414.12)	386.53 (326.92 to 458.11)	0.08 (-0.28 to 0.44)	0.673	6.18 (5.52 to 6.83)	6.31 (5.29 to 7.52)	0.04 (-0.33 to 0.41)	0.837
Peru	11.73 (8.71 to 15.62)	6.2 (4.32 to 8.53)	-2.1 (-2.18 to -2.02)	<0.001	287.78 (212.83 to 379.03)	141.42 (98.12 to 198.33)	-2.15 (-3.89 to -0.38)	0.017	4.56 (3.33 to 6.06)	2.22 (1.5 to 3.16)	-2.19 (-4.03 to -0.31)	0.023
Panama	7.52 (5.18 to 10.52)	4.33 (2.71 to 6.55)	-1.79 (-1.83 to -1.75)	<0.001	135.32 (116.16 to 156.36)	71.83 (56.99 to 88.95)	-2.11 (-2.93 to -1.28)	<0.001	2.13 (1.81 to 2.48)	1.11 (0.86 to 1.4)	-2.17 (-3.1 to -1.23)	<0.001
Portugal	9.05 (6.61 to 12.06)	2.46 (1.37 to 4.13)	-4.15 (-4.28 to -4.03)	<0.001	223.83 (195.95 to 254.42)	45.38 (38.84 to 52.45)	-5.12 (-5.89 to -4.34)	<0.001	3.57 (3.1 to 4.08)	0.65 (0.54 to 0.76)	-5.46 (-6.26 to -4.65)	<0.001
Palau	29.74 (22.41 to 39.28)	28.42 (22.2 to 36.2)	-0.18 (-0.25 to -0.1)	<0.001	775.07 (518.63 to 1101.74)	839.65 (593.69 to 1149.66)	0.19 (-0.15 to 0.52)	0.271	12.61 (8.24 to 18.19)	13.66 (9.5 to 18.96)	0.17 (-0.21 to 0.56)	0.377
Poland	8.13 (4.99 to 12.3)	3.37 (1.99 to 5.22)	-2.79 (-2.87 to -2.71)	<0.001	183.92 (172.58 to 195.63)	57.52 (51.7 to 63.82)	-3.67 (-4.46 to -2.86)	<0.001	2.92 (2.76 to 3.09)	0.83 (0.76 to 0.91)	-3.94 (-4.88 to -2.98)	<0.001
Puerto Rico	6.4 (4.28 to 9.28)	3.86 (2.28 to 6.07)	-1.58 (-1.71 to -1.45)	<0.001	83.51 (70.97 to 97.95)	38.36 (30.79 to 47.15)	-2.29 (-3.63 to -0.94)	0.001	1.3 (1.09 to 1.54)	0.54 (0.42 to 0.68)	-2.86 (-4.64 to -1.04)	0.002

Qatar	13.8 (10.09 to 18.7)	6.6 (4.31 to 9.63)	-2.35 (-2.48 to -2.21)	<0.001	224.1 (155.93 to 314.65)	77.21 (53.88 to 109.31)	-3.47 (-4.08 to -2.86)	<0.001	3.2 (2.09 to 4.66)	0.98 (0.62 to 1.5)	-3.79 (-4.43 to -3.16)	<0.001
Rwanda	33.64 (25.81 to 43.59)	9.86 (7.13 to 13.45)	-3.93 (-4.06 to -3.81)	<0.001	910.69 (621.15 to 1286.01)	226.36 (139.11 to 344.96)	-4.46 (-4.77 to -4.15)	<0.001	14.9 (9.99 to 21.16)	3.63 (2.17 to 5.65)	-4.51 (-4.81 to -4.21)	<0.001
Republic of Moldova	11.06 (8.36 to 14.55)	5.18 (3.59 to 7.33)	-2.47 (-2.76 to -2.19)	<0.001	231.02 (196.92 to 270.17)	98.23 (81.14 to 117.58)	-2.56 (-4.02 to -1.08)	0.001	3.76 (3.18 to 4.43)	1.54 (1.24 to 1.86)	-2.77 (-3.51 to -2.01)	<0.001
Romania	9.32 (6.96 to 12.42)	4.75 (3.28 to 6.79)	-2.18 (-2.23 to -2.13)	<0.001	205.45 (174.1 to 239.04)	97.18 (79.53 to 117.4)	-2.25 (-2.64 to -1.86)	<0.001	3.29 (2.77 to 3.87)	1.45 (1.17 to 1.79)	-2.42 (-2.88 to -1.96)	<0.001
Russian Federation	8.11 (4.98 to 12.22)	7.33 (4.95 to 10.35)	-0.35 (-0.64 to -0.06)	0.019	147.6 (140.92 to 154.17)	150.26 (136.05 to 162.21)	0.09 (-1.17 to 1.37)	0.887	2.35 (2.27 to 2.43)	2.47 (2.23 to 2.67)	0.2 (-1.09 to 1.51)	0.762
Republic of Korea	19.52 (14.05 to 26.36)	5.9 (3.57 to 9.21)	-3.82 (-3.97 to -3.68)	<0.001	299.99 (232.3 to 382.96)	59.92 (46.44 to 77.26)	-5.03 (-5.33 to -4.72)	<0.001	4.5 (3.35 to 5.85)	0.72 (0.54 to 0.99)	-5.69 (-6.04 to -5.35)	<0.001
Somalia	21.88 (16.22 to 29.16)	14.2 (10.81 to 18.64)	-1.41 (-1.48 to -1.35)	<0.001	538.4 (326.55 to 815.16)	347.46 (189.79 to 556)	-1.39 (-1.58 to -1.2)	<0.001	8.78 (5.19 to 13.47)	5.63 (2.96 to 9.16)	-1.41 (-1.57 to -1.24)	<0.001
South Sudan	15.29 (10.93 to 21.19)	8.66 (6.22 to 11.8)	-1.84 (-1.89 to -1.78)	<0.001	359.96 (232.93 to 525.95)	270.3 (163.57 to 425.44)	-0.83 (-1.21 to -0.44)	<0.001	5.8 (3.66 to 8.61)	4.34 (2.54 to 6.94)	-0.83 (-1.23 to -0.43)	<0.001
Sierra Leone	14.69 (10.25 to 20.53)	13.63 (9.92 to 18.41)	-0.27 (-0.33 to -0.21)	<0.001	375.85 (251.14 to 540.48)	348.51 (218.66 to 512.07)	-0.27 (-0.53 to -0.01)	0.045	5.93 (3.83 to 8.7)	5.49 (3.34 to 8.25)	-0.27 (-0.54 to 0)	0.05
Senegal	14.66 (10.37 to 19.85)	10.61 (7.5 to 14.51)	-1.04 (-1.12 to -0.95)	<0.001	432.9 (304.73 to 595.48)	254.42 (174.96 to 362.44)	-1.51 (-1.68 to -1.35)	<0.001	6.88 (4.76 to 9.61)	3.96 (2.64 to 5.77)	-1.56 (-1.73 to -1.38)	<0.001
Solomon Islands	35.33 (26.16 to 46.72)	37.3 (29.41 to 47)	0.15 (0.1 to 0.19)	<0.001	502.72 (274.35 to 739.07)	537.01 (363.6 to 758.36)	0.21 (0.01 to 0.4)	0.04	8.18 (4.14 to 12.27)	8.76 (5.71 to 12.54)	0.22 (0.01 to 0.43)	0.04
Sao Tome and Principe	18.54 (13.22 to 25.36)	15.85 (11.55 to 21.23)	-0.57 (-0.64 to -0.5)	<0.001	353.44 (214.15 to 511.24)	275.16 (166.83 to 438.86)	-0.83 (-1.3 to -0.36)	0.001	5.63 (3.29 to 8.27)	4.23 (2.39 to 7)	-0.93 (-1.41 to -0.44)	<0.001
Sudan	16.47 (11.96 to 22.02)	8.28 (5.98 to 11.09)	-2.24 (-2.34 to -2.14)	<0.001	608.95 (386.09 to 881.4)	272.21 (150.05 to 431.02)	-2.57 (-2.67 to -2.48)	<0.001	9.92 (6.19 to 14.46)	4.33 (2.28 to 6.99)	-2.65 (-2.79 to -2.51)	<0.001
Samoa	23.1 (17.04 to 30.78)	23.39 (17.76 to 30.42)	0.03 (-0.07 to 0.12)	0.617	561.06 (364.72 to 816.83)	566.27 (379.87 to 817.9)	0.02 (-0.02 to 0.06)	0.379	9.02 (5.7 to 13.39)	9.1 (5.85 to 13.46)	0.02 (-0.03 to 0.06)	0.445
Syrian Arab Republic	27.43 (21.62 to 34.68)	13.62 (10.48 to 17.39)	-2.24 (-2.37 to -2.12)	<0.001	795.72 (596.54 to 1036.07)	317.5 (227.89 to 444.21)	-2.87 (-3.32 to -2.42)	<0.001	12.45 (9.2 to 16.44)	4.85 (3.39 to 6.93)	-2.93 (-3.4 to -2.45)	<0.001
Suriname	13.58 (10.42 to 17.47)	9.42 (7.23 to 12.13)	-1.18 (-1.25 to -1.11)	<0.001	380.87 (257.93 to 497.04)	239.41 (169.64 to 329.98)	-1.26 (-2.89 to 0.4)	0.136	6.35 (4.27 to 8.33)	3.97 (2.77 to 5.52)	-1.55 (-3.54 to 0.47)	0.131
Saint Vincent and the Grenadines	10.99 (8.34 to 14.4)	6.63 (4.91 to 8.9)	-1.63 (-1.69 to -1.56)	<0.001	302.97 (258.78 to 355.13)	150.99 (123 to 183.44)	-2.23 (-3.04 to -1.4)	<0.001	5.03 (4.28 to 5.91)	2.49 (2.01 to 3.05)	-2.25 (-3.13 to -1.36)	<0.001
Saint Lucia	11.47 (8.59 to 15.36)	5.94 (4.15 to 8.27)	-2.11 (-2.16 to -2.06)	<0.001	259.21 (223.68 to 296.9)	106.72 (85.14 to 132.23)	-2.97 (-4.02 to -1.91)	<0.001	4.34 (3.74 to 5.01)	1.69 (1.33 to 2.13)	-3.15 (-4.24 to -2.05)	<0.001
South Africa	18.01 (11.88 to 25.89)	7.19 (4.85 to 10.2)	-2.98 (-3.15 to -2.8)	<0.001	487.84 (423.1 to 564.2)	202.49 (172.71 to 237.5)	-2.74 (-4.07 to -1.39)	<0.001	8.19 (7.06 to 9.49)	3.35 (2.85 to 3.94)	-2.82 (-4.05 to -1.57)	<0.001
Sri Lanka	10.55 (7.25 to 14.81)	6.42 (4.24 to 9.3)	-1.6 (-1.67 to -1.54)	<0.001	303.46 (234.65 to 390.26)	163.42 (108.92 to 230.89)	-1.95 (-3.55 to -0.32)	0.019	4.72 (3.57 to 6.17)	2.48 (1.55 to 3.63)	-2.01 (-3.74 to -0.25)	0.026
Seychelles	17.9 (13.3 to 23.64)	11.43 (8.34 to 15.51)	-1.46 (-1.63 to -1.29)	<0.001	401.16 (310.15 to 507.88)	188.44 (143.34 to 242.65)	-1.9 (-2.16 to -1.65)	<0.001	6.53 (4.97 to 8.4)	2.9 (2.16 to 3.8)	-2.07 (-2.35 to -1.79)	<0.001
Saint Kitts and Nevis	21.5 (17.12 to 26.97)	6.48 (4.4 to 9.15)	-3.87 (-4.27 to -3.47)	<0.001	514.82 (433.18 to 606.59)	86.24 (60.85 to 119.31)	-5.78 (-6.74 to -4.81)	<0.001	8.78 (7.36 to 10.37)	1.34 (0.9 to 1.91)	-6.05 (-7.08 to -5)	<0.001
Spain	6.72 (4.69 to 9.37)	2.33 (1.21 to 4)	-3.36 (-3.49 to -3.23)	<0.001	142.2 (123.39 to 162.35)	27.95 (23.74 to 32.45)	-5.41 (-6.02 to -4.79)	<0.001	2.17 (1.86 to 2.5)	0.36 (0.3 to 0.43)	-5.95 (-6.75 to -5.16)	<0.001
Serbia	9.78 (7.22 to 13.31)	3.94 (2.49 to 5.92)	-2.89 (-2.95 to -2.83)	<0.001	224.58 (170.06 to 290.8)	59.48 (43.53 to 78.5)	-4.2 (-4.55 to -3.85)	<0.001	3.55 (2.61 to 4.67)	0.81 (0.56 to 1.12)	-4.64 (-5.04 to -4.24)	<0.001
Slovakia	6.96 (4.77 to 9.93)	3.68 (2.2 to 5.72)	-2.04 (-2.11 to -1.97)	<0.001	151.98 (115.35 to 194.72)	51.01 (37.74 to 67.18)	-3.4 (-4.1 to -2.68)	<0.001	2.38 (1.76 to 3.11)	0.67 (0.47 to 0.94)	-3.88 (-4.57 to -3.19)	<0.001

Saudi Arabia	9.15 (6.53 to 12.52)	7.19 (5.27 to 9.68)	-0.77 (-0.89 to -0.66)	<0.001	344.71 (233.49 to 487.34)	224.34 (148.2 to 331.11)	-1.4 (-1.58 to -1.21)	<0.001	5.53 (3.69 to 7.93)	3.6 (2.29 to 5.43)	-1.39 (-1.59 to -1.19)	<0.001
Slovenia	6.13 (4.06 to 9.01)	2.66 (1.37 to 4.68)	-2.63 (-2.8 to -2.45)	<0.001	80.3 (67.07 to 95.91)	16.12 (12.11 to 20.62)	-5.11 (-5.87 to -4.36)	<0.001	1.15 (0.96 to 1.37)	0.11 (0.08 to 0.14)	-7.32 (-8.53 to -6.09)	<0.001
Singapore	9.11 (5.94 to 13.47)	4.27 (2.32 to 7.27)	-2.42 (-2.55 to -2.29)	<0.001	109.55 (94.5 to 126.02)	40.22 (32.98 to 48.37)	-3.12 (-4.02 to -2.21)	<0.001	1.56 (1.35 to 1.79)	0.46 (0.39 to 0.54)	-3.75 (-5.1 to -2.38)	<0.001
Sweden	3.15 (1.64 to 5.32)	2.77 (1.38 to 4.88)	-0.38 (-0.49 to -0.27)	<0.001	43.98 (37.55 to 50.98)	17.63 (13.9 to 22.27)	-2.99 (-3.4 to -2.58)	<0.001	0.61 (0.52 to 0.71)	0.15 (0.12 to 0.18)	-4.52 (-5.16 to -3.87)	<0.001
San Marino	3.19 (1.7 to 5.4)	2.75 (1.43 to 4.64)	-0.48 (-0.55 to -0.41)	<0.001	56.44 (40.83 to 75.63)	24.41 (16.45 to 34.14)	-2.8 (-3.04 to -2.57)	<0.001	0.84 (0.58 to 1.17)	0.29 (0.16 to 0.46)	-3.56 (-3.87 to -3.25)	<0.001
Switzerland	3.4 (1.88 to 5.67)	2 (0.88 to 3.74)	-1.67 (-1.88 to -1.47)	<0.001	57.25 (48.17 to 67.18)	12.62 (10.29 to 15.29)	-4.98 (-5.68 to -4.28)	<0.001	0.82 (0.68 to 0.98)	0.11 (0.09 to 0.14)	-6.41 (-7.48 to -5.33)	<0.001
Togo	16.41 (12.07 to 21.83)	12.91 (9.49 to 17.41)	-0.83 (-1.02 to -0.63)	<0.001	425.75 (293.68 to 576.73)	310.54 (193.83 to 455.74)	-0.97 (-1.23 to -0.72)	<0.001	6.81 (4.61 to 9.33)	4.94 (2.98 to 7.38)	-0.98 (-1.25 to -0.72)	<0.001
Timor-Leste	11.33 (7.78 to 16.02)	11.12 (7.99 to 15.27)	-0.04 (-0.21 to 0.13)	0.668	400.19 (276.38 to 553.26)	366.92 (210.87 to 545.13)	-0.4 (-1.05 to 0.26)	0.238	6.52 (4.41 to 9.11)	5.9 (3.25 to 8.94)	-0.44 (-1.1 to 0.23)	0.197
Tajikistan	12.18 (8.78 to 16.64)	6.4 (4.44 to 9.12)	-2.04 (-2.12 to -1.95)	<0.001	293.05 (220.33 to 380.15)	144.86 (96.98 to 206.86)	-2.27 (-2.88 to -1.67)	<0.001	4.75 (3.49 to 6.23)	2.27 (1.46 to 3.32)	-2.38 (-2.98 to -1.77)	<0.001
Tuvalu	30.98 (23.25 to 40.91)	27.11 (21.01 to 34.71)	-0.46 (-0.58 to -0.35)	<0.001	1021.56 (693.09 to 1421.6)	762.67 (540.09 to 1045.88)	-0.94 (-1.02 to -0.86)	<0.001	16.9 (11.23 to 23.77)	12.5 (8.73 to 17.34)	-0.97 (-1.04 to -0.89)	<0.001
Tonga	11.72 (8.26 to 16.22)	10.02 (7.16 to 13.74)	-0.5 (-0.56 to -0.43)	<0.001	230.25 (159.17 to 315.81)	224.49 (150.89 to 332.66)	-0.1 (-0.29 to 0.1)	0.33	3.58 (2.36 to 5.05)	3.45 (2.22 to 5.25)	-0.09 (-0.28 to 0.09)	0.307
Turkmenistan	12.38 (9.36 to 16.29)	14.88 (11.63 to 19.18)	0.65 (0.56 to 0.73)	<0.001	222.99 (185.52 to 266.49)	329.51 (253.95 to 424.85)	1.38 (0.19 to 2.58)	0.023	3.54 (2.91 to 4.28)	5.25 (3.96 to 6.87)	1.42 (-0.01 to 2.87)	0.052
Tunisia	8.16 (5.58 to 11.58)	5.63 (3.78 to 8.14)	-1.19 (-1.22 to -1.17)	<0.001	196.84 (133.17 to 275.88)	111.47 (71.15 to 165.02)	-1.85 (-1.99 to -1.7)	<0.001	3.07 (2.02 to 4.38)	1.67 (1 to 2.57)	-1.96 (-2.12 to -1.8)	<0.001
Thailand	15.3 (11.25 to 20.55)	12.69 (9.78 to 16.32)	-0.62 (-0.66 to -0.57)	<0.001	248.79 (175.06 to 337.54)	337.26 (235.28 to 465.27)	1.01 (-0.02 to 2.04)	0.054	3.78 (2.55 to 5.3)	5.48 (3.74 to 7.68)	1.24 (0.13 to 2.36)	0.029
Tokelau	21.75 (15.87 to 29.37)	16.04 (11.84 to 21.33)	-1.02 (-1.08 to -0.96)	<0.001	626.07 (393.95 to 966.5)	640.94 (445.46 to 861.25)	0.16 (-0.14 to 0.46)	0.292	10.23 (6.28 to 16.06)	10.23 (6.96 to 13.89)	0.09 (-0.21 to 0.39)	0.563
Turkey	13.8 (9.79 to 18.86)	5.84 (3.74 to 8.64)	-2.75 (-2.81 to -2.69)	<0.001	319.92 (230.05 to 433.46)	95.58 (69.1 to 128.5)	-3.84 (-4.5 to -3.19)	<0.001	4.96 (3.47 to 6.87)	1.35 (0.92 to 1.87)	-4.11 (-4.83 to -3.39)	<0.001
Trinidad and Tobago	8.81 (6.37 to 11.89)	4.89 (3.24 to 7.1)	-1.87 (-1.95 to -1.79)	<0.001	188.08 (162.4 to 217)	123.5 (91.66 to 161.91)	-1.25 (-2.29 to -0.2)	0.019	3.05 (2.61 to 3.52)	2.01 (1.46 to 2.68)	-1.2 (-2.33 to -0.06)	0.04
Taiwan (Province of China)	14.78 (10.59 to 20.02)	8.67 (5.7 to 12.38)	-1.72 (-1.78 to -1.66)	<0.001	240.6 (209.96 to 273.75)	108.77 (90.9 to 128.7)	-2.67 (-3.5 to -1.82)	<0.001	3.51 (3.07 to 4.01)	1.44 (1.23 to 1.68)	-2.99 (-4 to -1.96)	<0.001
Uganda	12.89 (9.5 to 17.39)	9.56 (7.12 to 12.7)	-0.94 (-1.11 to -0.76)	<0.001	310.73 (193.45 to 465.53)	217.05 (136.04 to 316.3)	-1.24 (-1.51 to -0.97)	<0.001	4.98 (3.03 to 7.56)	3.45 (2.08 to 5.13)	-1.27 (-1.55 to -0.99)	<0.001
United Republic of Tanzania	14.71 (10.93 to 19.38)	9.83 (7.22 to 13.18)	-1.3 (-1.33 to -1.27)	<0.001	333.23 (229.62 to 465.97)	201.61 (130.2 to 293.56)	-1.62 (-1.81 to -1.43)	<0.001	5.36 (3.63 to 7.58)	3.17 (1.98 to 4.7)	-1.69 (-1.88 to -1.49)	<0.001
Uzbekistan	12.08 (9.03 to 16.01)	9.57 (7.14 to 12.51)	-0.76 (-0.92 to -0.61)	<0.001	327.87 (290.43 to 366.66)	137.03 (113.85 to 161.9)	-2.87 (-4.77 to -0.93)	0.004	5.35 (4.73 to 6.01)	2.09 (1.71 to 2.5)	-3.07 (-5.17 to -0.93)	0.005
Uruguay	10.6 (7.3 to 14.87)	5.31 (3.25 to 8.22)	-2.22 (-2.28 to -2.16)	<0.001	194.07 (169.6 to 221.53)	72.83 (62.48 to 85)	-3.22 (-3.47 to -2.97)	<0.001	3.02 (2.63 to 3.47)	1.01 (0.86 to 1.18)	-3.55 (-3.84 to -3.25)	<0.001
Ukraine	8.3 (5.08 to 12.63)	10.12 (6.84 to 14.57)	0.68 (0.34 to 1.03)	<0.001	123.17 (102.1 to 146.85)	141.71 (102.61 to 186.42)	0.25 (-1.09 to 1.6)	0.719	1.87 (1.51 to 2.24)	2.24 (1.57 to 3.01)	0.36 (-1.06 to 1.79)	0.623
United States Virgin Islands	6.77 (4.49 to 9.89)	4.14 (2.48 to 6.52)	-1.54 (-1.82 to -1.26)	<0.001	154.24 (104.2 to 218.4)	107.91 (63.22 to 173.08)	-0.57 (-1.37 to 0.23)	0.161	2.53 (1.67 to 3.63)	1.71 (0.95 to 2.79)	-0.69 (-1.54 to 0.16)	0.112
United Arab Emirates	9.98 (7.03 to 13.91)	6.85 (4.66 to 9.82)	-1.2 (-1.3 to -1.1)	<0.001	194.41 (130.99 to 282.16)	103.31 (70.38 to 144.18)	-1.78 (-3.49 to -0.04)	0.045	2.77 (1.77 to 4.18)	1.41 (0.88 to 2.06)	-1.89 (-3.81 to 0.08)	0.059

United Kingdom	3.73<br>(2.07 to 6.11)	2.64<br>(1.44 to 4.44)	-1.11<br>(-1.2 to -1.02)	<0.001	48.7<br>(45.44 to 52.53)	24.24<br>(21.81 to 27.05)	-2.37<br>(-3.19 to -1.54)	<0.001	0.68<br>(0.66 to 0.7)	0.3<br>(0.29 to 0.32)	-2.72<br>(-3.71 to -1.72)	<0.001
United States of America	4.03<br>(2.23 to 6.54)	3.16<br>(1.8 to 5.04)	-0.76<br>(-0.94 to -0.57)	<0.001	64.36<br>(59.51 to 70.06)	47.72<br>(43 to 53.28)	-0.93<br>(-1.34 to -0.52)	<0.001	0.9<br>(0.86 to 0.94)	0.63<br>(0.58 to 0.68)	-1.12<br>(-1.76 to -0.47)	0.001
Vanuatu	29.13<br>(21.62 to 38.78)	32.61<br>(25.54 to 41.16)	0.36<br>(0.27 to 0.44)	<0.001	854.15<br>(561.87 to 1238.42)	853.11<br>(560.39 to 1198.23)	-0.03<br>(-0.21 to 0.15)	0.738	14.01<br>(9.02 to 20.57)	13.9<br>(8.88 to 19.84)	-0.05<br>(-0.23 to 0.13)	0.603
Venezuela (Bolivarian Republic of)	9.42<br>(6.76 to 12.9)	6.3<br>(4.42 to 8.71)	-1.29<br>(-1.42 to -1.17)	<0.001	186.16<br>(160.11 to 215.65)	130.17<br>(94.05 to 169.51)	-0.97<br>(-1.56 to -0.36)	0.002	3.01<br>(2.56 to 3.51)	2.08<br>(1.46 to 2.76)	-0.98<br>(-1.54 to -0.42)	0.001
Viet Nam	14.63<br>(10.71 to 19.62)	10.91<br>(8 to 14.69)	-0.97<br>(-1.11 to -0.83)	<0.001	377.4<br>(265.34 to 525.03)	243.68<br>(167.82 to 352.66)	-1.43<br>(-1.55 to -1.31)	<0.001	6.23<br>(4.25 to 8.79)	3.88<br>(2.58 to 5.78)	-1.54<br>(-1.66 to -1.42)	<0.001
Yemen	14.13<br>(10.23 to 19.14)	8.37<br>(6.21 to 11.1)	-1.69<br>(-1.71 to -1.67)	<0.001	404.37<br>(213.35 to 629.11)	236.66<br>(138.17 to 365.54)	-1.61<br>(-2.25 to -0.97)	<0.001	6.74<br>(3.46 to 10.58)	3.85<br>(2.18 to 6.02)	-1.71<br>(-2.22 to -1.21)	<0.001
Zimbabwe	5.05<br>(3.3 to 7.36)	6.04<br>(4.34 to 8.19)	0.57<br>(0.42 to 0.73)	<0.001	104.23<br>(72.87 to 145.55)	279.07<br>(177.49 to 424.05)	3.29<br>(2.58 to 4)	<0.001	1.6<br>(1.07 to 2.28)	4.5<br>(2.81 to 6.95)	3.31<br>(2 to 4.64)	<0.001
Zambia	17.47<br>(13.34 to 22.72)	11.43<br>(8.77 to 14.88)	-1.4<br>(-1.47 to -1.32)	<0.001	453.88<br>(314.51 to 629.89)	302.57<br>(186.08 to 461.35)	-1.31<br>(-1.48 to -1.14)	<0.001	7.4<br>(5.06 to 10.35)	4.92<br>(2.96 to 7.57)	-1.31<br>(-1.49 to -1.14)	<0.001

Supplementary Table 4 The predictive analysis of the DALYs rate and cases of intracerebral hemorrhage among adolescents and young adults populations in global from 1990 to 2021

Year	Both			Male			Female		
	ASR	Crude rate	cases	ASR	Crude rate	cases	ASR	Crude rate	cases
2022	174.63	178.56	5429416	219.24	223.39	3448283	129.18	132.08	1977413
2023	172.45	176.57	5394798	217.12	221.46	3437640	126.93	129.97	1953555
2024	170.26	174.44	5352121	215.00	219.34	3421795	124.68	127.77	1926880
2025	168.52	172.64	5316623	212.97	217.14	3403036	123.23	126.32	1910425
2026	166.77	170.73	5276600	210.93	214.82	3381751	121.77	124.79	1892272
2027	165.03	168.73	5233736	208.90	212.42	3359279	120.32	123.18	1872876
2028	163.28	166.61	5185523	206.87	209.91	3334131	118.86	121.48	1851348
2029	161.54	164.38	5131498	204.83	207.29	3305806	117.40	119.69	1827639
2030	160.42	162.77	5096187	203.42	205.30	3287173	116.55	118.50	1812830
2031	159.30	161.24	5063751	202.01	203.42	3270566	115.69	117.36	1798903
2032	158.18	159.84	5038862	200.60	201.73	3258784	114.84	116.30	1787539
2033	157.06	158.65	5019052	199.19	200.35	3250596	113.99	115.35	1777653
2034	155.94	157.54	5002024	197.77	199.10	3244562	113.13	114.43	1768538
2035	155.19	156.85	4998508	196.71	198.27	3245255	112.67	113.94	1766118
2036	154.45	156.22	4995906	195.65	197.51	3246260	112.21	113.48	1764103
2037	153.70	155.62	4993462	194.58	196.79	3246798	111.75	113.05	1762362
2038	152.96	155.05	4990310	193.52	196.08	3246128	111.29	112.65	1760697
2039	152.21	154.52	4986938	192.46	195.41	3244836	110.82	112.27	1759131
2040	152.07	154.66	5003365	192.30	195.73	3258370	110.66	112.23	1762442
2041	151.93	154.84	5018911	192.14	196.09	3271099	110.50	112.22	1765518
2042	151.79	155.04	5033395	191.98	196.48	3282994	110.34	112.23	1768261
2043	151.65	155.26	5046424	191.82	196.87	3293553	110.18	112.25	1770659
2044	151.51	155.51	5058372	191.66	197.30	3303019	110.02	112.30	1772828

Supplementary Table 5 The predictive analysis of the deaths rate and cases of intracerebral hemorrhage among adolescents and young adults populations in global from 1990 to 2021

Year	Both			Male			Female		
	ASR	Crude rate	cases	ASR	Crude rate	cases	ASR	Crude rate	cases
2022	2.75	2.83	85954	3.54	3.62	55841	1.94	1.99	29854
2023	2.72	2.79	85393	3.50	3.58	55625	1.90	1.96	29446
2024	2.68	2.76	84692	3.46	3.55	55316	1.86	1.92	28990
2025	2.65	2.73	84111	3.43	3.51	54968	1.84	1.90	28730
2026	2.62	2.70	83444	3.39	3.47	54571	1.82	1.88	28438
2027	2.60	2.67	82719	3.36	3.42	54148	1.79	1.85	28121
2028	2.57	2.63	81890	3.32	3.38	53673	1.77	1.82	27762
2029	2.54	2.59	80955	3.28	3.33	53139	1.75	1.79	27364
2030	2.52	2.57	80334	3.26	3.30	52781	1.73	1.77	27123
2031	2.50	2.54	79764	3.24	3.26	52459	1.72	1.75	26897
2032	2.48	2.52	79328	3.21	3.23	52224	1.71	1.74	26714
2033	2.47	2.50	78994	3.19	3.21	52061	1.70	1.72	26563
2034	2.45	2.48	78713	3.16	3.19	51939	1.68	1.71	26426
2035	2.44	2.47	78630	3.14	3.17	51923	1.68	1.70	26392
2036	2.42	2.46	78567	3.12	3.16	51916	1.67	1.70	26366
2037	2.41	2.45	78511	3.10	3.15	51903	1.66	1.69	26347
2038	2.40	2.44	78448	3.09	3.13	51872	1.66	1.68	26332
2039	2.38	2.43	78387	3.07	3.12	51833	1.65	1.68	26322
2040	2.38	2.43	78663	3.06	3.13	52052	1.64	1.68	26337
2041	2.38	2.44	78929	3.06	3.13	52260	1.64	1.67	26351
2042	2.38	2.44	79182	3.06	3.14	52456	1.63	1.67	26362
2043	2.37	2.44	79415	3.05	3.15	52632	1.63	1.67	26369
2044	2.37	2.45	79636	3.05	3.15	52793	1.62	1.67	26375

Supplementary Table 6 The predictive analysis of the incidence rate and cases of intracerebral hemorrhage among adolescents and young adults populations in global from 1990 to 2021

Year	Both			Male			Female		
	ASR	Crude rate	cases	ASR	Crude rate	cases	ASR	Crude rate	cases
2022	8.25	8.43	256210	9.92	10.10	155917	6.54	6.68	100082
2023	8.24	8.42	257357	9.89	10.08	156527	6.54	6.69	100599
2024	8.22	8.41	258151	9.87	10.06	156917	6.54	6.70	100983
2025	8.22	8.41	258848	9.84	10.03	157181	6.55	6.71	101414
2026	8.21	8.39	259352	9.82	9.99	157339	6.56	6.71	101771
2027	8.20	8.37	259741	9.80	9.96	157448	6.57	6.71	102079
2028	8.19	8.35	259888	9.77	9.91	157439	6.58	6.71	102287
2029	8.19	8.32	259761	9.75	9.86	157287	6.59	6.70	102382
2030	8.18	8.29	259694	9.74	9.83	157347	6.59	6.69	102368
2031	8.18	8.27	259771	9.73	9.80	157506	6.59	6.68	102402
2032	8.18	8.25	260228	9.72	9.77	157898	6.60	6.67	102579
2033	8.17	8.25	260938	9.71	9.77	158460	6.60	6.67	102838
2034	8.17	8.25	261797	9.70	9.76	159131	6.60	6.67	103143
2035	8.17	8.25	262781	9.70	9.77	159980	6.60	6.67	103409
2036	8.16	8.25	263817	9.70	9.79	160851	6.60	6.67	103700
2037	8.16	8.25	264868	9.70	9.80	161707	6.60	6.67	104009
2038	8.16	8.26	265894	9.69	9.82	162516	6.60	6.67	104324
2039	8.15	8.27	266916	9.69	9.83	163304	6.60	6.68	104647
2040	8.17	8.30	268414	9.70	9.86	164198	6.62	6.70	105267
2041	8.18	8.33	269866	9.70	9.89	165053	6.63	6.73	105873
2042	8.19	8.36	271264	9.71	9.93	165868	6.65	6.76	106460
2043	8.20	8.39	272583	9.71	9.96	166615	6.67	6.78	107025
2044	8.21	8.42	273845	9.72	9.99	167309	6.69	6.81	107575
